# Linking diet to growth, nutrient composition, and flavor characteristics in Chinese mitten crab (*Eriocheir sinensis*): a study based on biochemical composition and intestinal microbiota

**DOI:** 10.3389/fnut.2026.1798709

**Published:** 2026-04-01

**Authors:** Gang Jiang, Xingwei Du, Wenqiang Ren, Xueyan Ma, Qing Wang, Yongkai Tang

**Affiliations:** 1 Key Laboratory of Freshwater Fisheries and Germplasm Resources Utilization, Ministry of Agriculture and Rural Affairs, Freshwater Fisheries Research Center, Chinese Academy of Fishery Sciences, Wuxi, China; 2 Changshu Station for Popularization of Fisheries Technology, Changshu, China; 3 Wuxi Fisheries College, Nanjing Agricultural University, Wuxi, China

**Keywords:** dietary supplementation, *Eriocheir sinensis*, gut microbiota, muscle quality, nutrient composition, volatile compounds

## Abstract

**Background:**

The search for sustainable alternatives to fish meal in aquafeeds is critical for the aquaculture industry. However, the impact of alternative protein sources on the comprehensive quality of Chinese mitten crab (Eriocheir sinensis) remains underexplored.

**Methods:**

This study investigated the effects of dietary supplementation with beef liver (BL), housefly maggot (HM), or frozen trash fish (FTF) on the comprehensive quality of *E. sinensis*.

**Conclusion:**

Our results highlight that BL and FTF, in particular, confer distinct and significant advantages. Results indicated that BL and HM significantly enhanced growth performance, while FTF had no adverse effects. BL supplementation notably increased muscle crude protein, essential amino acids, and key minerals (K, Fe, Cu). FTF supplementation led to the highest deposition of PUFAs, including EPA and DHA. Volatile compound analysis revealed that dietary regimens distinctively shaped the flavor profile. Partial least squares-discriminant analysis identified 15 key flavor markers, with 2-Butanone (M) (imparting fruity and cereal notes) being the most significant. Furthermore, BL and FTF modulated the gut microbiota and enhanced antioxidant capacity. Mechanistically, these quality improvements are primarily attributed to the modulation of nutrient deposition, lipid oxidation, and amino acid metabolism, likely mediated by shifts in the intestinal microbiota. These findings demonstrate that BL and FTF are effective dietary strategies for improving the muscle quality, flavor, and health of *E. sinensis*, offering promising and sustainable alternatives to conventional feeds.

## 1 Introduction

The Chinese mitten crab (*Eriocheir sinensis*) is a commercially significant freshwater crustacean in China. It is highly valued by consumers for its desirable sensory qualities and rich nutritional content, particularly in high-quality proteins, lipids, essential amino acids, and various micronutrients. In 2023, the nation production yield reached 888,629 tons (1, 2). Research has established that the nutritional composition of muscle in aquaculture species is influenced by a combination of genetic, dietary, and environmental factors, including germplasm, feed formulation, and culture practices (3–5). Nowadays, food safety concept and people's requirement for high-quality aquatic products have become stringent increasingly (6), optimizing the feed formula for river crabs is of great significance for improving their growth and enhancing the quality of their muscle.

In intensive culture systems, the river crab is predominantly fed commercial aquafeeds. Historically, these feeds have utilized fish meal (FM) and fish oil (FO)- derived from finite wild marine fisheries- as principal sources of protein and lipids (7–9). However, the sustainability of this reliance is threatened by escalating global, fluctuating costs, and ecological concerns regarding wild fish stock depletion (10, 11). These challenges necessitate the search for sustainable alternative ingredients to ensure the long-term viability of aquaculture (12–14). The Chinese mitten crab possesses an omnivorous feeding habit, naturally consuming a diverse spectrum of organisms such as molluscs, worms, crustaceans, and detritus (15, 16). This physiological adaptability suggests a considerable potential for the partial or complete substitution of FM and FO in its diet with alternative, economically viable, and nutritionally adequate protein and lipid sources. Such a dietary shift is crucial for enhancing the sustainability of the industry without compromising the final product quality.

Indeed, a wide range of alternative feed ingredients- such as terrestrial plants, animal by-products, insects, microbial biomass, macroalgae, and food wastes—have been investigated to reduce reliance on FM and FO in aquafeeds (17, 18, 81, 86). Among these, housefly (*Musca domestica*) maggot meal (HM), frozen trash fish (FTF) and beef liver (BL) have emerged as particularly promising animal-derived alternatives due to their high nutritional value and availability (19, 20, 82–84). However, current research on their application in crustacean aquaculture, especially in crabs, remains fragmented and has yielded inconsistent findings.

HM is rich in protein (40–58.5 %) and lipids (23–34 %), with a balanced essential amino acid (EAA) profile, and diverse micronutrients (21, 82, 85). While studies have demonstrated its potential to enhance growth and disease resistance in some aquatic species, its effects on flesh quality, particularly flavor and nutrient deposition, are less documented and sometimes controversial (79, 80). Similarly, FTF, primarily composed of small, low – value marine fish, is widely used in crab hatcheries due to its high levels of protein and unsaturated fatty acids, especially EPA and DHA (22–24). Despite its common use, concerns persist regarding its variable quality, potential for introducing pathogens, and inconsistent effects on final product quality (21, 25, 26, 78). BL, another animal-derived feed, is nutritionally dense, contains substantial levels of L-carnitine, glutathione, vitamin A and E, as well as essential minerals like iron and zinc (27, 28). Although some farmers have adopted BL as a supplement or replacement for formulated feeds, scientific evidence supporting its efficacy in crab aquaculture, particularly regarding its impact on muscle quality and flavor, is scarce.

Despite the individual merits of these alternative feeds, a critical knowledge gap remains: no systematic comparison has been conducted to evaluate how different animal-derived protein sources (HM, FTF, and BL) holistically affect the comprehensive quality of crabs. Specifically, their differential impacts on key quality traits—such as muscle nutritional composition, volatile flavor compounds, and the underlying regulatory mechanisms involving intestinal microbiota and host metabolism—have not been rigorously investigated. Previous studies have primarily focused on growth performance or single nutritional indices, overlooking the integrated effects on overall product quality and the complex biological interactions that drive these changes. Therefore, a comprehensive evaluation that simultaneously examines growth, nutritional composition, flavor characteristics, and intestinal microbial dynamics is urgently needed to provide a scientific basis for optimizing feed formulations and improving crab quality.

This study comprehensively assessed the effects of four feeds—HM, FTF, BL, and CF—on the growth performance, physiological indices, nutritional quality, trace element content, and volatile organic compound (VOC) profiles of crabs. By integrating flavor analysis with nutritional measurements, we characterized the overall quality of crabs fed different diets. Furthermore, intestinal microbiome sequencing and metabolomic analysis of crab intestinal tissues were performed to further elucidate the potential mechanisms by which these feeds influence crab quality. The findings provide a theoretical foundation for healthy crab cultivation and offer new insights for optimizing feed formulations and improving product quality, thereby supporting the sustainable development of the crab aquaculture industry.

## 2 Materials and methods

### 2.1 Ethical statement

This study was conducted in accordance with the institutional guidelines for animal care and use for scientific purposes, following review and approval by the Animal Ethics Committee of the Freshwater Fisheries Research Center, Chinese Academy of Fishery Sciences (Wuxi, China) (Protocol LAECFFRC-2023-06-12).

### 2.2 Experimental materials and design

The culturing experiment was conducted at collaborative breeding bases in Changshu City, Suzhou, Jiangsu Province. A total of 12 ponds were used, each with an area of 20,000 m^2^. Juvenile crabs (20.44 ± 0.81 g) were stocked at a density of 1,200 individuals per 667 m^2^. In February, all ponds were uniformly renovated and disinfected, and water depth was standardized. *Elodea nuttallii* was densely planted at 1 m intervals, with each bundle measuring 10 cm in size. To minimize environmental heterogeneity and ensure the comparability of replicate ponds within each treatment group, rigorous standardization measures were implemented across all 12 ponds throughout the experimental period, including shared water source, synchronized water quality management, consistent stocking sources and densities, and standardized feeding protocols (detailed below).

Four feeding strategies were assigned evenly across the 12 ponds: the control group (treatment CF) fed with commercial-feed only (crude protein: 41 ± 0.5 %; crude lipid, 7.3 ± 0.5 %; ash, 9.7 ± 0.5 %; crude fiber, 5.0 ± 0.5 %; and moisture, 9.5 ± 0.5 %), treatment BL+CF fed with 80 % frozen beef liver and 20 % commercial-feed, treatment HM+CF fed with 80 % housefly Musca domestica maggot meal and 20 % commercial-feed, and treatment FTF+CF fed with 80 % frozen trash fish (small anchovies) and 20 % commercial-feed. The daily feeding rate was maintained at approximately 5% of the crab standing biomass. The trial lasted for 60 days, from April 1 to June 30, 2025. Throughout the experiment, water quality parameters were monitored regularly to ensure they met farming standards for Chinese mitten crab. Key parameters included: dissolved oxygen levels ≥5 mg/L, pH: 7.0–8.5, ammonia nitrogen (${\text{NH}}_{4}^{+}$-N): < 0.2 mg/L, and nitrite (${\text{NO}}_{2}^{-}$-N): < 0.1 mg/L.

### 2.3 Sample collection

At the end of the feeding trail, all groups underwent a 24-h fasting period prior to final sampling. A total of 30 crabs were randomly selected from each pond. This relatively large sample size per ponds (30 individuals × 3 ponds × 4 treatments = 360 crabs total for growth measurements) was chosen to obtain robust and representative estimates of each pond's mean growth performance, to capture the natural range of individual variation within each population, and to provide sufficient tissue for subsequent comprehensive analyses of nutrient composition, volatile compounds, mineral elements, and gut microbiota. After measuring growth parameters (body weight, body length, body width and body thickness), hemolymph was sampled from crab leg joints via a 1 mL syringe, and used for antioxidant activities assays. Crab was subsequently anesthetized on ice, and the intact hepatopancreas, muscle and intestines were extracted, immediately frozen in liquid nitrogen, and transferred to −80 °C for subsequent analysis. The body weight gain (BWG), body length gain (BLG), specific growth rate (SGR), and meat yield rate (MYR) of the experimental crabs are calculated using the following formulas:

Body weight gain (BWG, %) = [(final weight–initial weight)/initial weight] × 100%

Body length gain (BLG, %) = [(final length–initial length)/initial length] × 100%

Specific growth rate (SGR, % day ^−1^) = [(Ln final weight –Ln initial weight)/duration] × 100%

Meat yield rate (MYR, %) = (muscle weight/body weight) × 100%

### 2.4 Assay of antioxidant and immune related enzymes activity

The hemolymph samples were incubated for 2 h at 4 °C, and then centrifuged at 4,500 rpm for 15 min at 4 °C. The serum was separated and stored at −80 °C for superoxide dismutase (SOD), catalase (CAT), malondialdehyde (MDA), total antioxidant capacity (T-AOC), lysozyme (LZM), acid phosphatase (ACP), alkaline phosphatase (AKP) assays.

The hepatopancreas were dissected out, weighted and homogenized with saline solution (NaCl 28.4 g/L, MgCl·6H_2_O 1 g/L, MgSO_4_ ·7H_2_O_2_ g/L, CaCl_2_ ·2H_2_O_2_. 25 g/L, KCl 0.7 g/L, glucose 1 g/L, Hepes 2.38 g/L) in ice-cooled condition. After centrifugation at 4,500 rpm and 4 °C for 20 min, the supernatant fluid was collected for measurement of digestive enzyme activities. SOD, CAT, MDA, T-AOC, LZM, ACP, AKP, amylase and lipase activities were determined using kits (Jiancheng Biotech Co., Nanjing, China) according to the manufacturer's instructions. For the protease activity, the enzyme assays used casein as the substrate and Folin phenol as the reagent as per the method of Sigma's non-specific protease activity assay (29).

### 2.5 Histopathological analysis

Intestinal tissue samples (*n* = 5) were fixed in 4% paraformaldehyde (Solarbio, Beijing, China), rinsed with PBS, and subsequently processed for paraffin embedding. Briefly, the fixed tissues were washed under running water for 4 h, dehydrated through a graded ethanol series (70%, 80%, 90%, and 100%), embedded in paraffin, and sectioned at a thickness of 5 μm. Finally, the sections were stained with hematoxylin and eosin (H&E) for histological examination (30).

### 2.6 Muscle nutrient composition

#### 2.6.1 Measurement of basic nutrient contents

Muscle tissue specimens were obtained from five crabs in each group and processed according to established protocols of the Association of Official Analytical Chemists (31). Moisture was determined by drying samples in an oven at 105 °C until constant mass was achieved. Ash content was measured by combustion in a muffle furnace maintained at 550 °C for a duration of 6 h. Crude protein levels were quantified using the Kjeldahl technique (FOSS, Denmark), implemented with a 2300 Auto-analyzer. Total lipid extraction was performed individually via chloroform–methanol (2:1, v/v) mixture supplemented with 0.01% butylated hydroxytoluene (BHT) as an antioxidant, following the procedure outlined by Cejas et al. (32).

#### 2.6.2 Determination of fatty acid composition

The fatty acid composition of muscle tissue was analyzed using an Agilent 7890 Gas Chromatograph (Agilent, United States) coupled with a mass spectrometer (GC–MS). Separation was achieved on an SP™-2560 fused silica capillary column (100 m × 0.25 mm × 0.2 μm) following a procedure adapted from Wang et al. (17). As previously noted, total lipids were extracted from individual samples and subsequently transmethylated to fatty acid methyl esters using 14% boron trifluoride in methanol, according to the methodology of Morrison and Smith (33). Fatty acid peaks were identified by comparison with reference standards, and their relative contents were quantified based on the normalized peak area percentage.

#### 2.6.3 Amino acid content determination

The amino acid composition of muscle tissue was analyzed in accordance with the Chinese national food safety standard GB5009.124-2016, “Determination of amino acids in foods.” An acid hydrolysis procedure was employed to quantify amino acids, with the exception of tryptophan, which is degraded under acidic hydrolysis conditions. Briefly, approximately 100 mg of sample was accurately weighed into a hydrolysis tube, mixed with 8 mL of hydrochloric acid solution, and purged with nitrogen before sealing. The sealed tube was incubated at 120 °C for 22–24 h. After hydrolysis, the mixture was transferred to a volumetric flask, neutralized with 4.8 mL of sodium hydroxide (10 mol/L), and brought to a final volume of 25 mL with distilled water. The solution was subsequently filtered and centrifuged, and 400 μL of the supernatant was analyzed using an Agilent 1100 high-performance liquid chromatography (HPLC) system (Agilent, Santa Clara, CA, USA). Quantification of amino acids was performed based on chromatographic peak areas. Amino acid scores (AAS) and chemical scores (CS) were calculated using the FAO/WHO reference amino acid pattern and the whole egg protein amino acid profile, respectively, according to the following equations:

AAS = Amino acid content in sample (mg/g N)/Amino acid content in FAO scoring pattern (mg/g N)

CS = Amino acid content in sample (mg/g N)/Content of the same amino acid in whole egg protein (mg/g N)

### 2.7 Trace element determination

The concentrations of sodium (Na), potassium (K), calcium (Ca), iron (Fe), and copper (Cu) in muscle tissue (*n* = 5) were quantified using inductively coupled plasma–mass spectrometry (ICP-MS), following the procedures outlined in Method 1 of the national standard GB5009.268-2016. Specifically, a 0.5 g sample aliquot was transferred to a microwave digestion vessel, treated with nitric acid, and subjected to digestion in a constant-temperature drying oven. After digestion, the solution was diluted to a final volume of 50 mL and subsequently analyzed via ICP-MS to determine the levels of trace elements.

### 2.8 GC-IMS analysis

VOCs were analyzed using a FlavorSpec^®^ gas chromatography-ion mobility spectrometry (GC-IMS) system (GAS, Dortmund, Germany). Approximately 2 g of homogenized muscle was weighed into a 20 mL headspace vial, which was sealed and incubated at 45 °C for 20 min. Subsequently, 500 μL of the headspace gas was injected into the GC inlet (injection needle temperature: 85 °C).

Separation was performed on an MXT-5 column (15 m × 0.53 mm, 1.00 μm) held at 60 °C. High-purity nitrogen (≥99.999%) was used as both carrier and drift gas. The carrier gas flow rate was set as follows: 2 mL/min for 2 min, increased to 10 mL/min over 8 min, and finally raised to 100 mL/min for 10 min. The IMS conditions were set as follows: drift tube temperature, 45 °C; linear voltage, 500 V/cm; and drift gas flow, 150 mL/min.

Each sample was analyzed in triplicate. VOCs were identified by comparing the retention index and drift time against the NIST and IMS databases using LAV software (v. 2.2.1). Spectral data visualization, including two-dimensional plots, three-dimensional spectra, difference maps, and fingerprint profiles, was performed using the Reporter and Gallery Plot plug-ins.

### 2.9 16S rRNA sequencing in intestinal microbiota

Total genomic DNA was isolated from intestinal content samples (*n* = 5) using the E.Z.N.A.^®^ Soil DNA Kit (Omega Bio-tek, Norcross, GA, USA). DNA quality and integrity were evaluated with a NanoDrop 2000 spectrophotometer and agarose gel electrophoresis, respectively. The 16S rRNA gene was subjected to high-throughput sequencing by Shanghai Majorbio Biomedical Technology Co., Ltd. (Shanghai, China). The experimental workflow consisted of the following steps: extracted genomic DNA was first verified by 1% agarose gel electrophoresis. PCR amplification was performed with TransStart FastPfu DNA Polymerase (TransGen AP221-02) on an ABI GeneAmp^®^ 9700 system under standardized conditions, with triplicate reactions per sample. Amplified products were purified and assessed for purity, followed by quantification and integrity analysis using an Agilent 5400 automated capillary electrophoresis system. After library construction, quality checks were performed prior to Illumina sequencing. Raw reads were processed by assembly, denoising, and chimera filtering. High-quality sequences were clustered into operational taxonomic units (OTUs) at a 97% similarity threshold. Subsequent bioinformatic analyses included α-diversity assessment, community heterogeneity evaluation, and co-occurrence network construction.

Dependent acquisition mode was employed to collect data throughout the analysis process.

### 2.10 Data analysis

All experimental data were subjected to one-way ANOVA in SPSS 27.0 (IBM Corp., USA), followed by Duncan's multiple range test to determine significant differences among groups (*P* < 0.05). To control for the risk of false positives due to multiple comparisons, the Bonferroni correction was applied to adjust the significance level in *post-hoc* pairwise comparisons following one-way ANOVA for fatty acid and amino acid contents. A corrected *P*-value < 0.05 was considered statistically significant. Results are presented as mean ± SD. For fatty acid, amino acid, and volatile organic compound (VOC) profiles, and multivariate statistical analyses—principal component analysis (PCA) were performed via MetaboAnalyst v.5.0. Correlation analyses and visualizations, including cluster heatmaps and radar plots, were carried out using the Lianchuan Bioanalytics Cloud Platform (https://www.omicstudio.cn/home) and GraphPad Prism 9.0, respectively.

## 3 Results

### 3.1 Growth performance and nutrient contents

**Figure 1:**
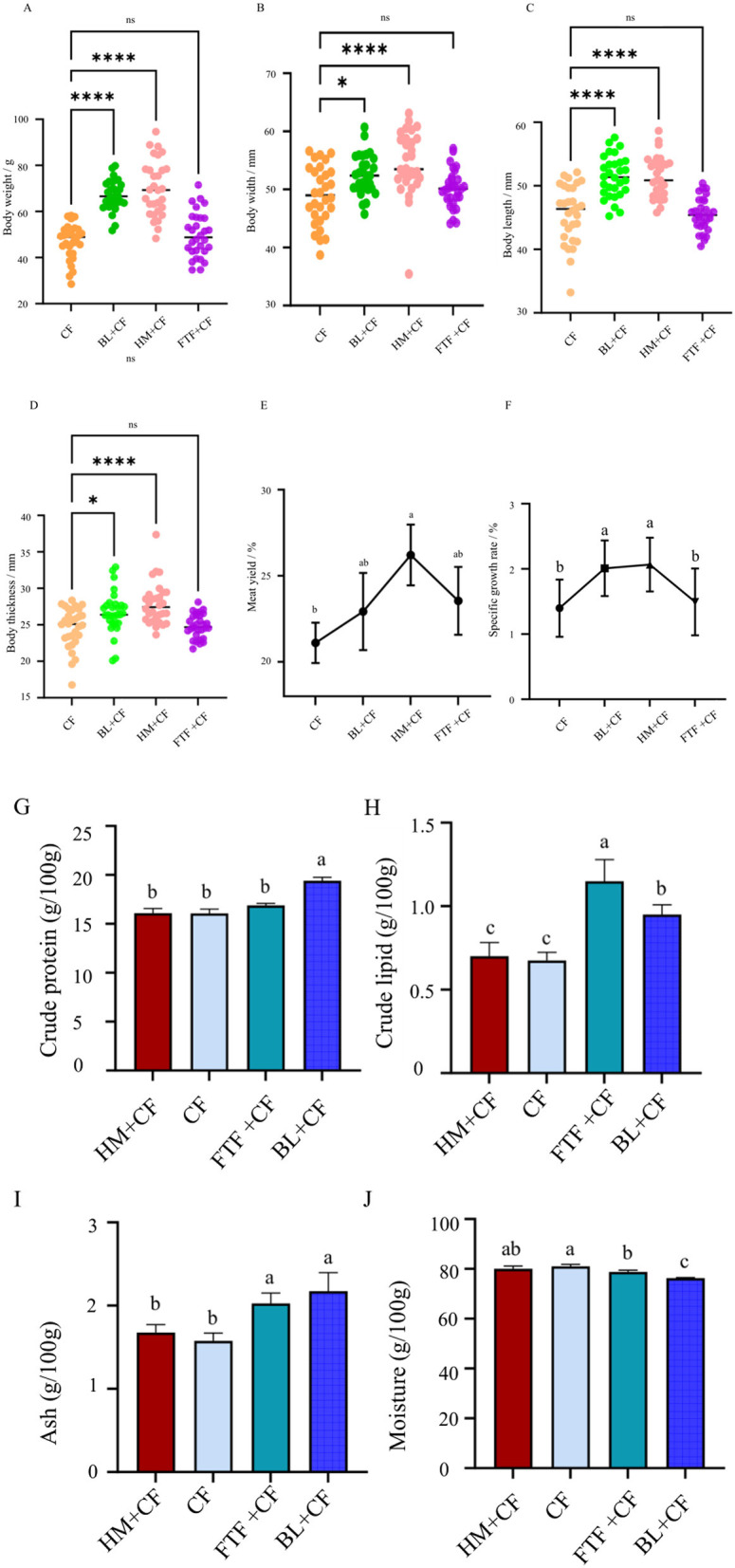
Growth performance and basic nutrient contents in muscle of *E. sinensis*. **(A)** Body weight; **(B)** Body width; **(C)** Body length; **(D)** Body thickness; **(E)** Meat yield; **(F)** SGR, specific growth rate; **(G)** Crude protein; **(H)** Crude lipid; **(I)** Ash; **(J)** Moisture. Different lowercase letters indicate significant differences between groups (*P* < 0.05). ^*^represents *P* < 0.05; ^***^represents *P* < 0.0001.

Dietary supplementation with the FTF did not significantly affect the growth performance of *E. sinensis* when compared with the control group fed CF alone (*P* > 0.05; Figures 1A–F). In contrast, diets in which CF was partially replaced by BL or HM significantly enhanced multiple growth parameters, including body length, weight, body width, body thickness, SGR and even meat yield (Figures 1A–F; *P* < 0.05).

Regarding muscle nutrition contents, the BL-supplemented group showed significantly higher crude protein and ash contents than other groups (Figure 1G, I; *P* < 0.05). Conversely, crude fat content was highest in the FTF group, followed by the BL group (Figure 1H; *P* < 0.05). No significant differences were observed in the proximate composition of crab muscle between the HM-supplemented group and the control (CF) group (Figures 1G–J; *P* > 0.05).

### 3.2 Analysis of serum biochemical indicators

**Figure 2:**
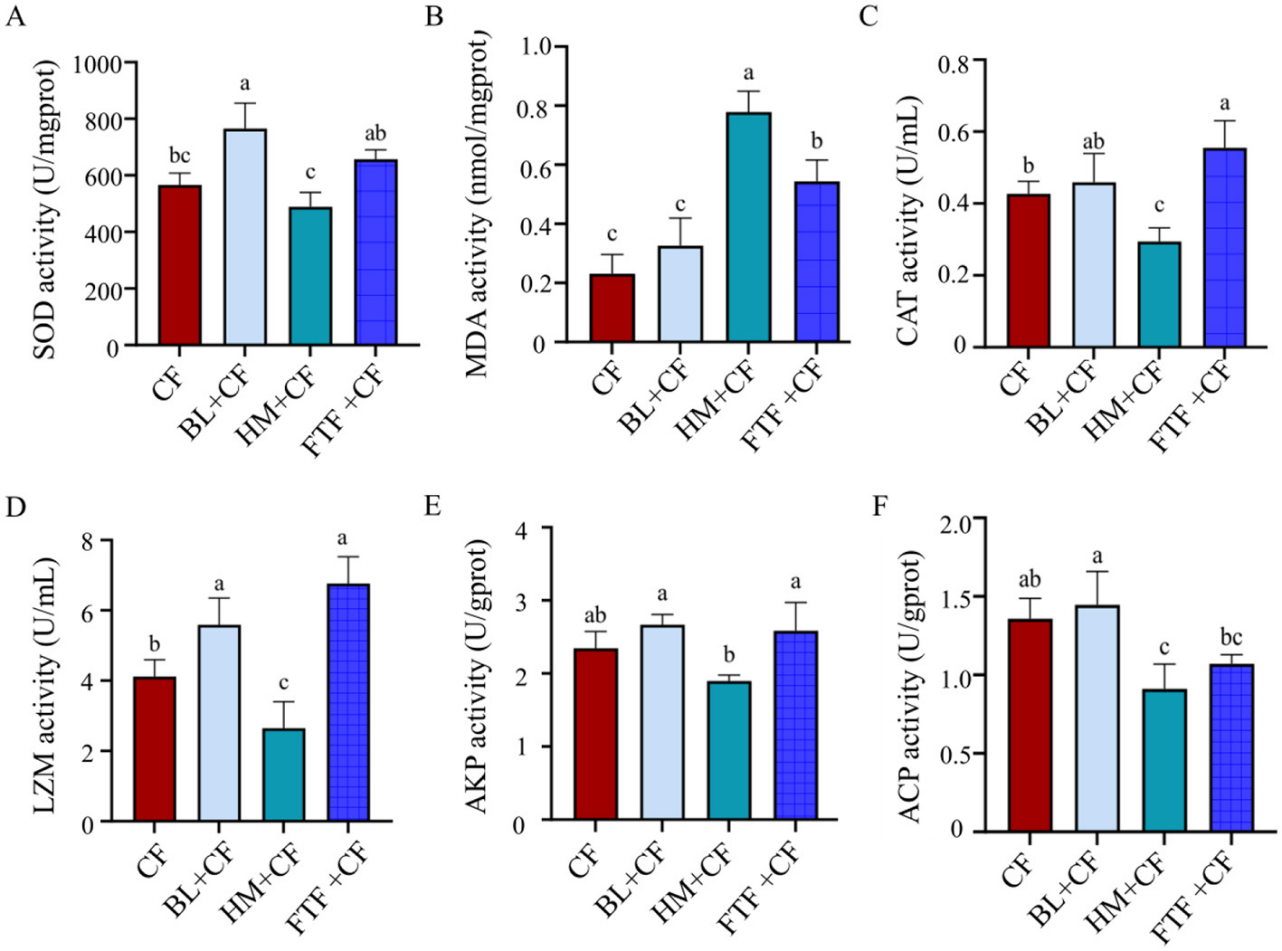
Serum antioxidant levels of *E. sinensis*. **(A)** SOD activity; **(B)** MDA activity; **(C)** CAT activity; **(D)** LZM activity; **(E)** AKP activity; **(F)** ACP activity. Different lowercase letters indicate significant differences between groups (*P* < 0.05).

Compared with the control group (CF), dietary supplementation with the HM or FTF could significantly increase the increased the activities of SOD, CAT, LZM and AKP values for crabs (Figures 2A, C–E; *P* < 0.05). However, the HM-supplemented group exhibited the highest MDA activity among all groups (Figure 2B; *P* < 0.05), while showing the lowest ACP activity (Figure 2F; *P* < 0.05).

### 3.3 Fatty acid composition and content analysis

**Figure 3:**
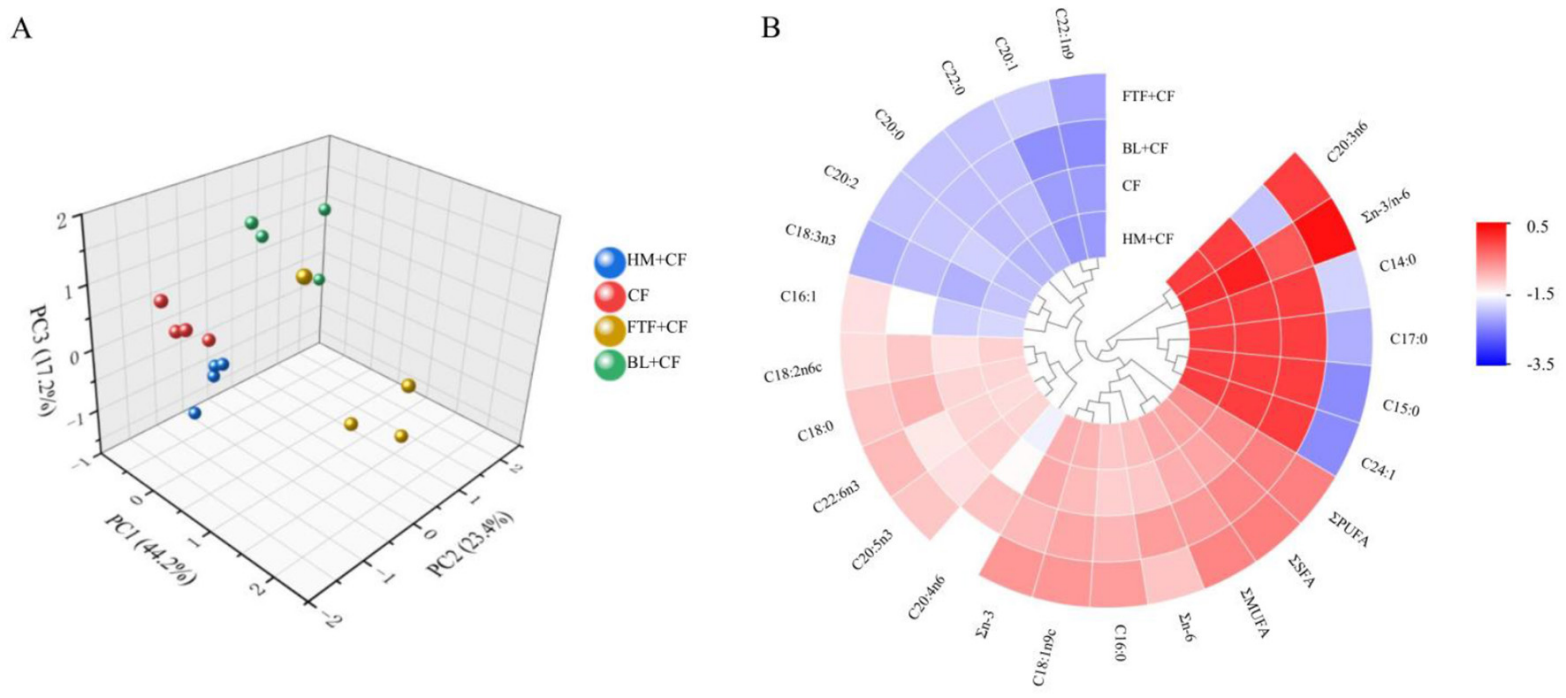
Comparison of fatty acids in muscle of *E. sinensis* among control and different experimental groups. **(A)** PCA plot based on fatty acid content; **(B)** Clustered heat map of fatty acid content.

The fatty acid composition of crab muscle was analyzed across the control and experimental groups. As presented in Supplementary Table S1, a total of 14 fatty acids were identified across all groups, categorized as four saturated fatty acids (SFA), four monounsaturated fatty acids (MUFA), and six polyunsaturated fatty acids (PUFA). Notably, specific fatty acids including C14:0, C15:0, C17:0, and C24:1 were exclusively identified in the FTF+CF group, while C20:3n6 was unique to the BL+CF treatment. Principal component analysis (PCA) demonstrated that the first two principal components (PCA1 and PCA2) collectively explained 67.6% of the total variance in fatty acid composition (Figure 3A), A distinct clustering was observed, with the FC+CF and BL+CF groups forming one cluster, whereas the CF and HM+CF groups exhibited no significant clustering.

To account for the multiple comparisons inherent in subsequent pairwise testing across 14 fatty acids, Bonferroni correction was applied, and statistical significance was determined at adjusted *P* < 0.05. *Post-hoc* comparisons based on this corrected threshold confirmed that the contents of ∑SFA, ∑MUFA, and ∑PUFA—particularly eicosapentaenoic acid (EPA) and docosahexaenoic acid (DHA)—, were significantly elevated in the FTF+CF group compared to all others (*P* < 0.05), with the BL+CF group ranking second. No significant differences were found between the CF and HM+CF groups (*P* > 0.05). Similarity, the Σn-3 PUFA content and the Σn-3/Σn-6 ratio was also highest in the FTF+CF group (*P* < 0.05). In contrast, the ∑n-6 content was significantly greater in the BL+CF group than in the others (*P* < 0.05), with no significant differences observed among the remaining groups (*P* > 0.05; Figure 3B). For individual fatty acids, the CF and HM+CF groups showed selective differences: linoleic acid (C18:2n6c, LA) and arachidonic acid (C20:4n6, ARA) were significantly different between these two groups after correction (adjusted *P* < 0.05), while most other individual fatty acids and all summary indices (∑SFA, ∑MUFA, ∑PUFA, ∑n-3, ∑n-6, ∑n-3/∑n-6) showed no significant differences between CF and HM+CF (adjusted *P* > 0.05). Individual fatty acid comparisons that remained significant after Bonferroni correction are indicated in Supplementary Table S1 with their adjusted *P*-values.

### 3.4 Amino acid composition and content analysis

**Figure 4:**
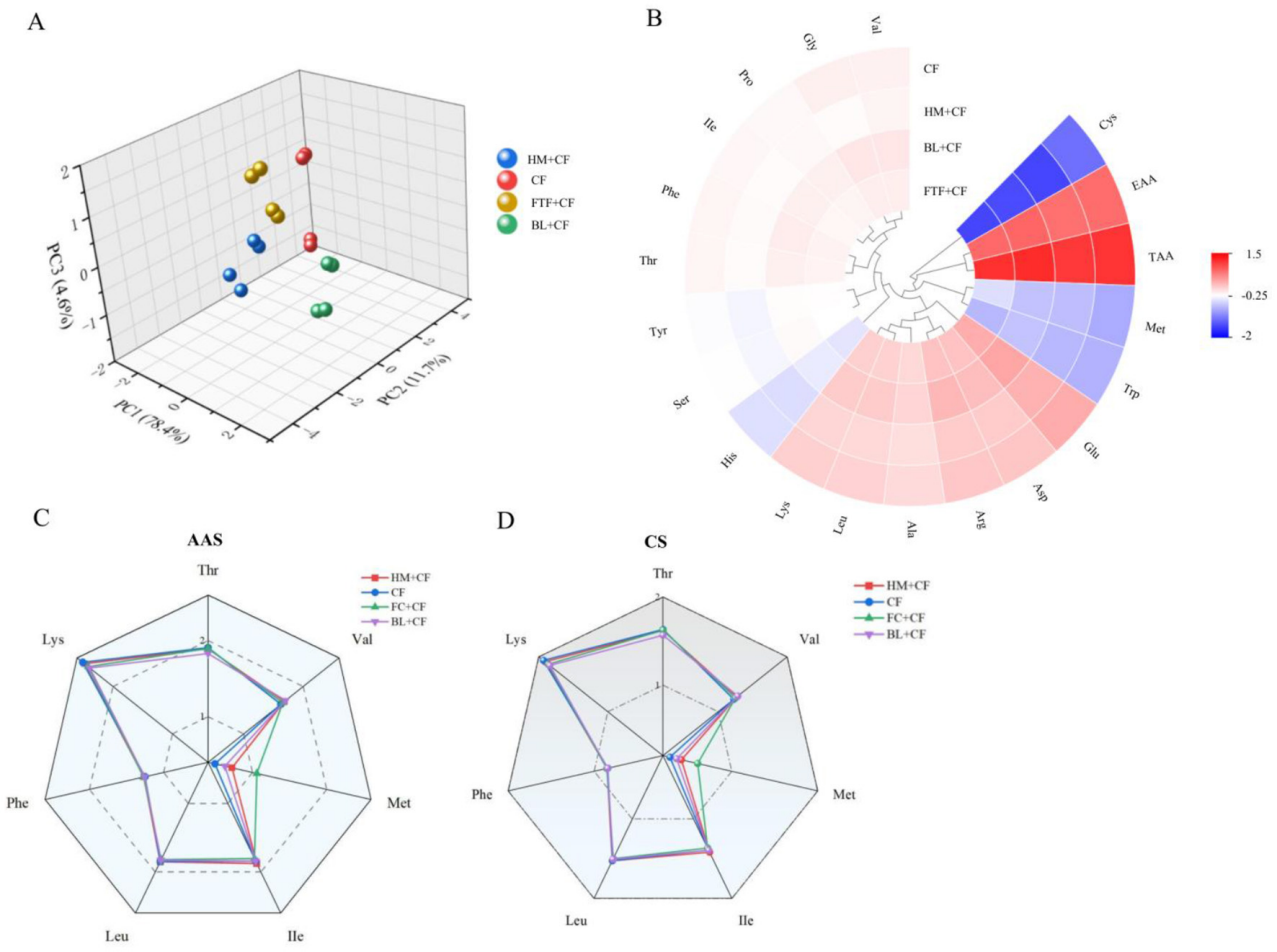
Comparison of amino acids in muscle of *E. sinensis* among control and different experimental groups. **(A)** PCA plot based on amino acids content; **(B)** Clustered heat map of amino acids content; **(C)** AAS score; **(D)** CS score.

Muscle amino acid analysis identified identified 18 amino acids across all groups, comprising 8 essential (EAA), 2 semi-essential, and 8 non-essential. Principal component analysis (PCA) revealed that the first two principal components (PCA1 and PCA2) accounted for 78.4% and 11.7%, respectively, of the total variance in amino acid composition (Figure 4A).

To address multiple comparisons across 18 amino acids and summary indices, Bonferroni correction was applied (adjusted *P* < 0.05). After correction, the BL+CF group exhibited a significantly enhanced amino acid profile. With the exception of methionine (Met), the concentrations of all other 17 amino acids were significantly higher in the BL+CF group compared to the CF control group (*P* < 0.05, Supplementary Table S2). Notably, Tryptophan (Trp), Met, Histidine (His) and Proline (Pro) were present at minimal levels in the CF group. The HM+CF group exhibited significantly lower levels of multiple amino acids, particularly aspartic acid (Asp), glutamic acid (Glu), and leucine (Leu) (*P* < 0.05). All summary measures (EAA, DAA, TAA) remained significantly highest in the BL+CF group after Bonferroni correction (*P* < 0.05). Individual amino acid comparisons that remained significant after correction are indicated in Supplementary Table S2 with their adjusted *P*-values.

A clustered heatmap analysis further corroborated the superior and distinct amino acid profile of the BL+CF group relative to all other treatments (Figure 4B). Consequently, the total amino acid content across groups followed the descending order: BL+CF > FTF+CF > CF > HM+CF, indicating that BL supplementation yielded the highest overall amino acid nutritional quality in crab muscle.

Notably, both AAS and CS evaluations in the present study identified Val as the primary limiting amino acid across all dietary groups (Figures 4C, D).

### 3.5 Comparison of mineral element contents

**Figure 5:**
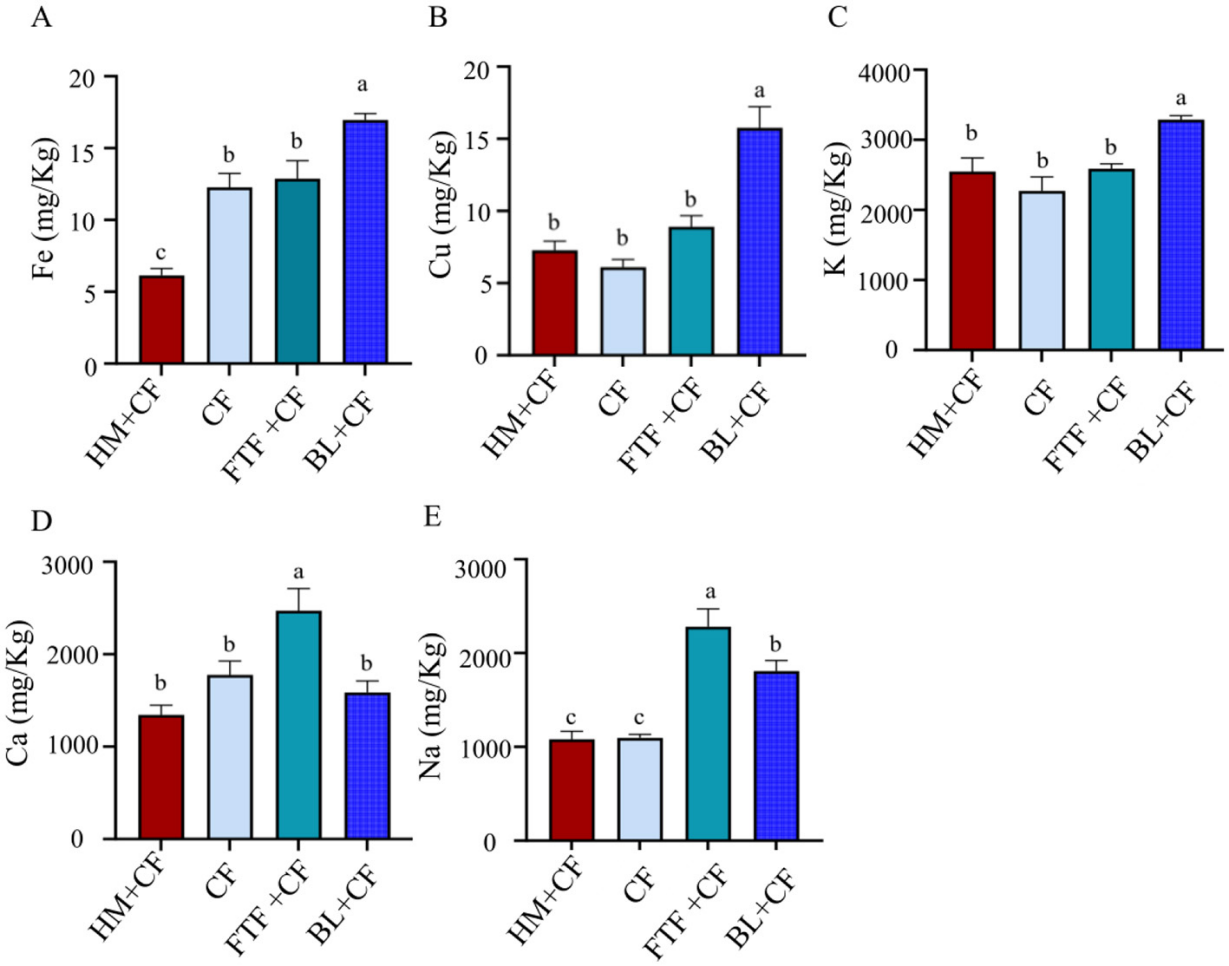
Comparison of mineral elements in muscle of *E. sinensis* among control and different experimental groups. **(A)** Fe content; **(B)** Cu content; **(C)** K content; **(D)** Ca content; **(E)** Na content; Different lowercase letters indicate significant differences between groups (*P* < 0.05).

The mineral composition of crab muscle was significantly influenced by dietary treatment (Figures 5A–E). Crabs fed the BL+CF diet accumulated significantly higher levels of potassium (K), iron (Fe), and copper (Cu) compared to all other groups (*P* < 0.05). Sodium (Na) content was highest in the FTF+CF group, followed by the BL+CF group; with both being significantly greater than in the HM+CF and CF groups (*P* < 0.05). In contrast, the HM+CF and CF groups exhibited the lowest concentrations of Cu, K, calcium (Ca), and Na, with no significant differences these two groups (*P* > 0.05).

### 3.6 Volatile compound analysis

#### 3.6.1 Volatile compound variance analysis

**Figure 6:**
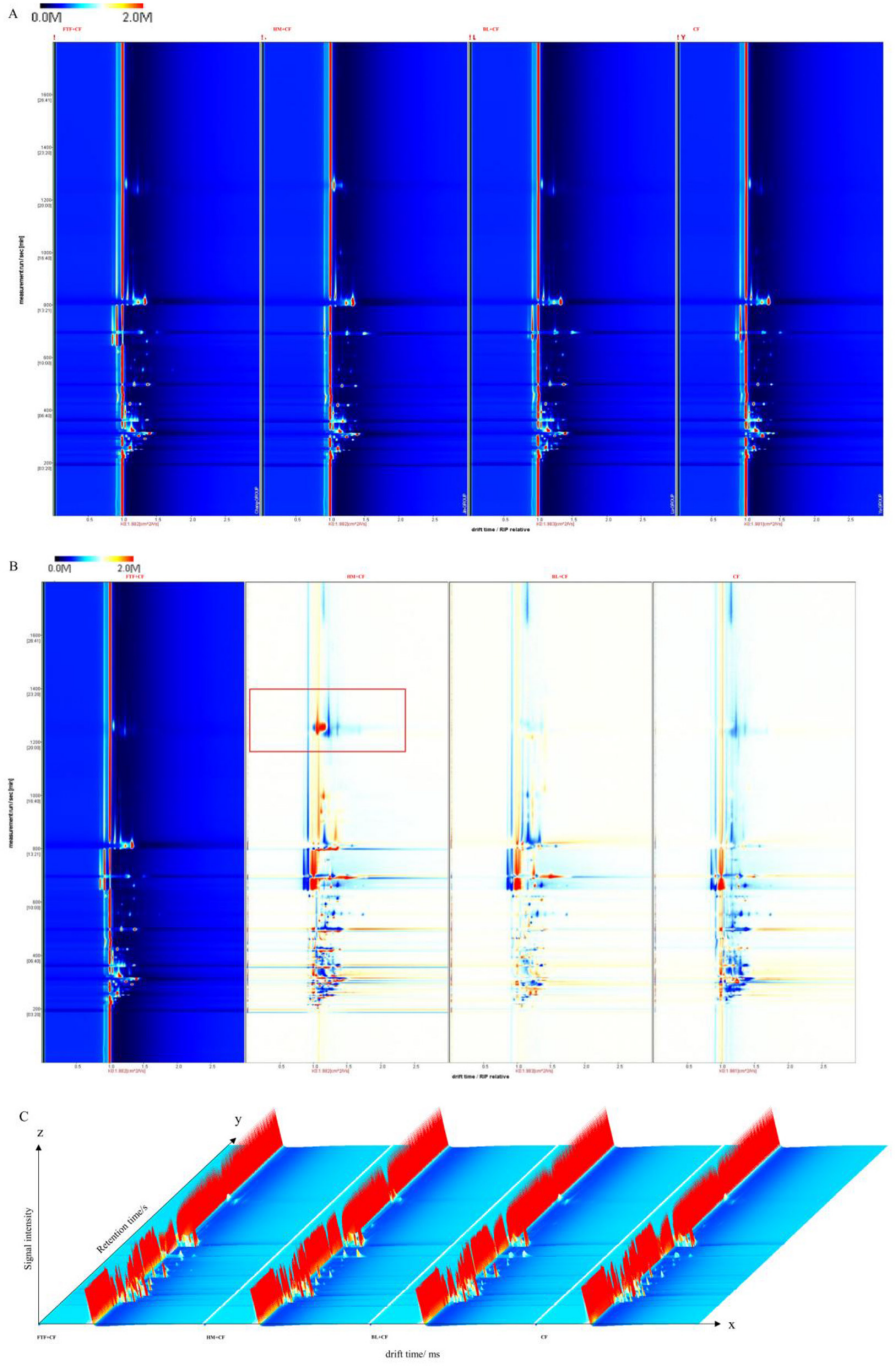
Results of GC-IMS analyses of the muscle of *E. sinensis* in control and experimental groups. **(A)** 2D top view mapping of VOCs; **(B)** 2D differential comparison mapping of VOCs; **(C)** 3D ion mobility mapping of VOCs.

The differential volatile profiles in muscle samples from the control and treatment groups were characterized using GC-IMS. Two-dimensional (2D) topographic visualization was conducted with the Reporter plug-in (Figure 6A). T In this map, the horizontal axis represents gas chromatographic retention time, and the vertical axis represents ion migration time, with a normalized reactive ion peak (RIP) marked at 1.0 ms. Signal intensity, indicated by a color gradient from red (high) to white (low), corresponds to the relative concentration of volatile organic compounds (VOCs). Most signals were concentrated within a migration time range of 0.5–1.5 ms and a retention time range of 1–8 min. Visibly distinct signal patterns among the dietary groups indicated substantial differences in their volatile compositions.

To directly compare these differences, a differential topographic map was constructed using the control group (CF) as the reference (Figure 6B). In this map, red regions signify higher concentrations, blue regions indicate lower concentrations, and white areas denote comparable levels relative to the reference. Notably, the HM+CF group exhibited prominent red signals, demonstrating a significant increase in both the diversity and abundance of specific VOCs compared to the control. These findings were further corroborated by three-dimensional ion mobility representations of the VOCs, which showed consistent group-wise variations (Figure 6C). Collectively, the GC-IMS analysis confirmed that dietary regimen substantially altered the volatile flavor profile of *E. sinensis* muscle.

#### 3.6.2 Identification of volatile compounds and fingerprint analysis

**Figure 7:**
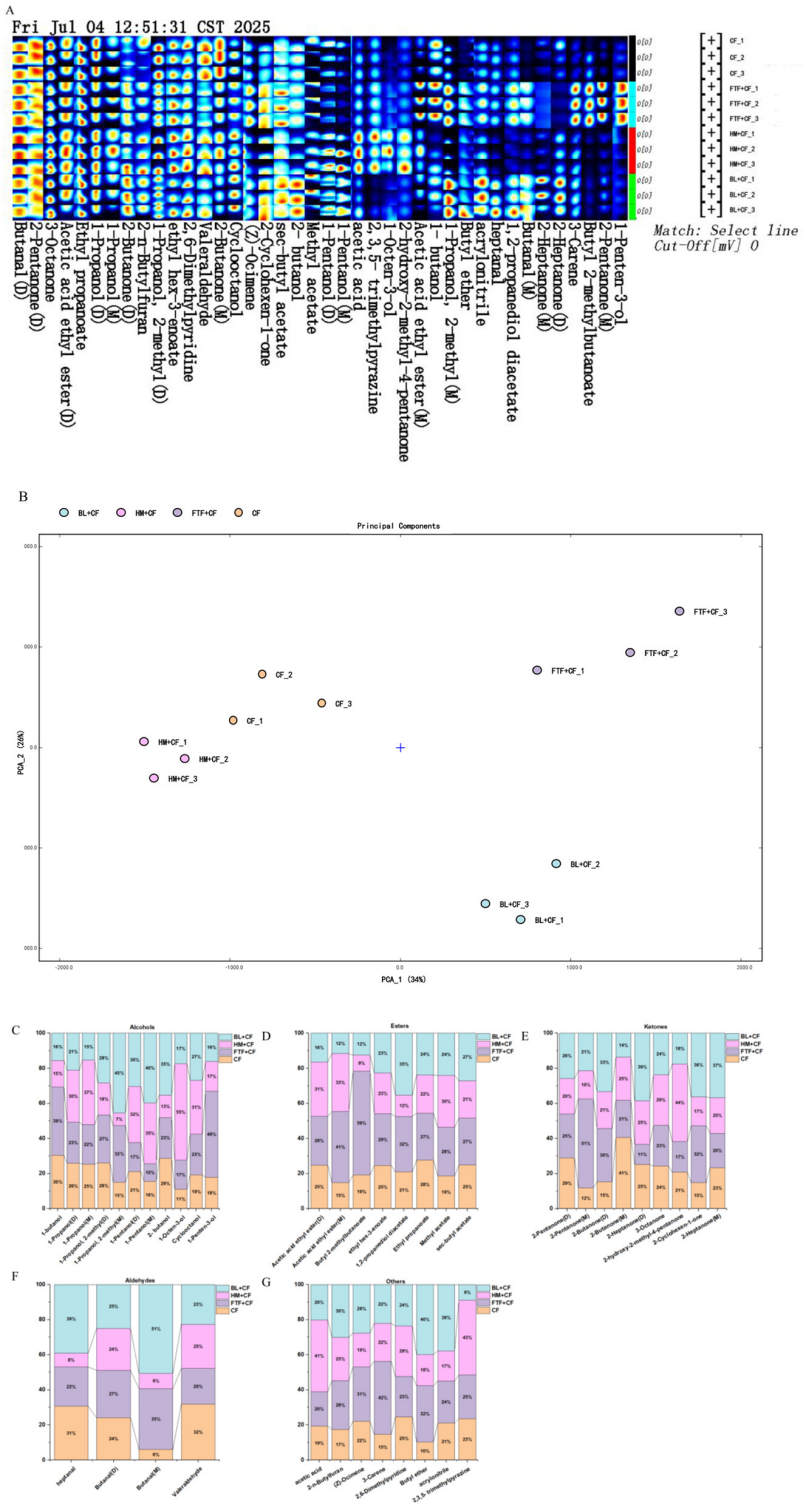
Types and contents of volatile compounds of *E. sinensis* in control and experimental groups. **(A)** Fingerprints of volatile compounds; **(B)** PCA analysis of volatile compounds; **(C–G)** Proportions of compounds in various classes in different groups. **(C)** Alcohols; **(D)** Esters; **(E)** Ketones; **(F)** Other compounds; **(G)** Aldehydes.

VOC profiling via GC-IMS Gallery Plot analysis identified 40 distinct signals across all dietary groups (Figure 7A). Six compounds generated multiple peaks due to dimer or multimer formation in the ionization region. Compared to the control (CF) group, each treatment exhibited a unique enhancement in specific VOCs. The FTF + CF group showed marked increases in compounds including acetic acid ethyl ester (M), 1-butanoic acid, 1-propanoic acid, 2-methyl (M), 3-carene, butyric acid 2-methylbutanoate, 2-pentanone (M), and 1-penten-3-one. The HM+CF group displayed elevated levels of acetic acid, 2,3,5-trimethylpyrazine, 1-octen-3-one, and 2-hydroxy-2-methyl ester (M), while the BL + CF group featured higher abundances of 1-propanoic acid, acrylonitrile, and 2-heptanone (D). The 40 signals corresponded to a diverse array of VOCs, dominated by alcohols (11 compounds), followed by ketones (9), esters (8), aldehydes (4), and a heterogeneous group of 8 other compounds comprising furans, monoterpenes, pyridines, ethers, carboxylic acids, nitriles, and pyrazines.

**Figure 8:**
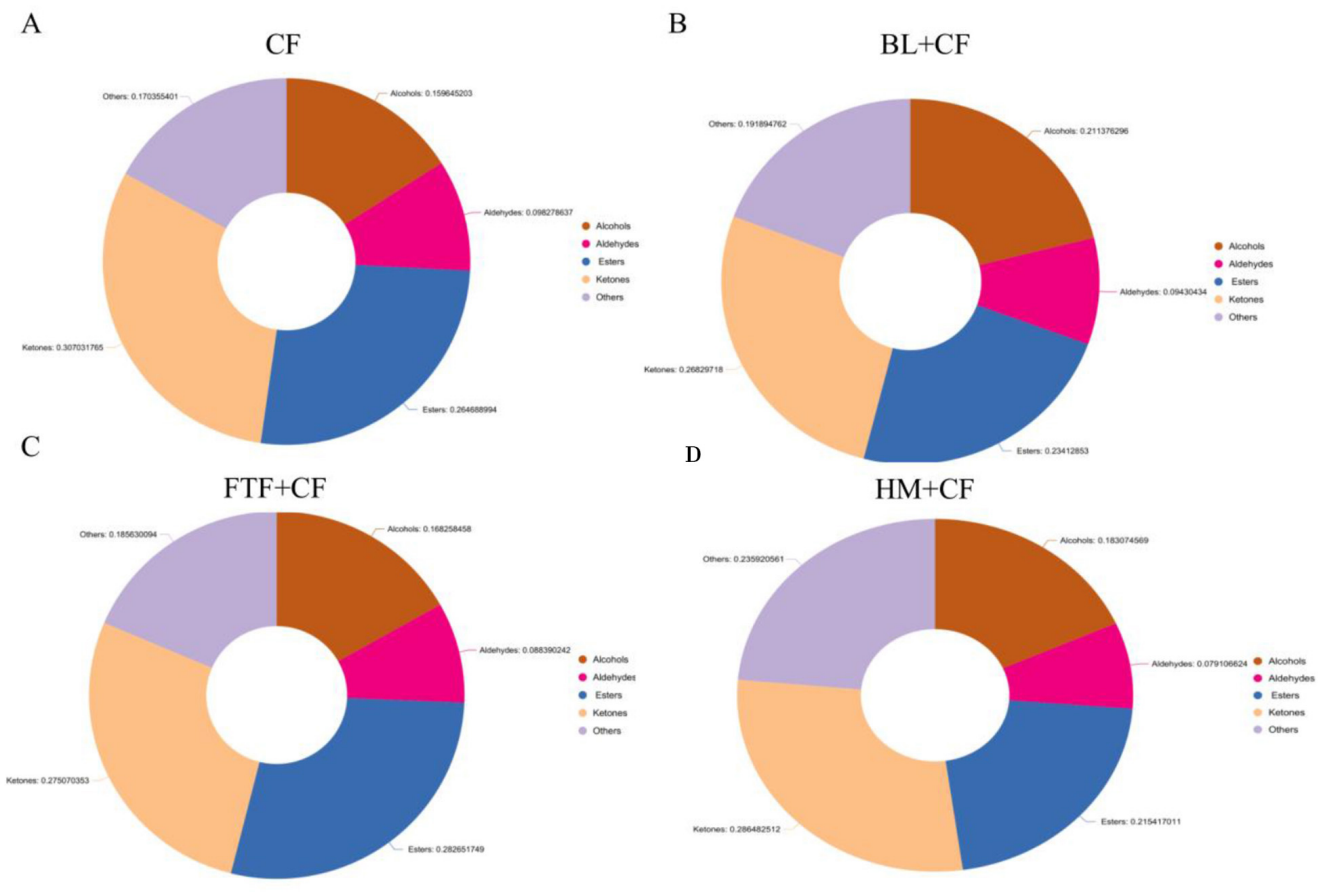
Proportions of VOCs (with flavor markers identified) in experimental groups and the control group. **(A–D)** Percentage of VOCs in the four different experimental groups.

PCA of the VOC profiles revealed clear separation among the four dietary groups (Figure 7B), confirming that dietary regimen significantly shaped the volatile composition. Statistical comparisons further detailed these differences (Figures 7C–G). Among the 11 significantly varied alcohols, 1-butanol, 1-propanol (D), 1-propanol (M), 1-octen-3-ol, cyclooctanol, and 11-penten-3-ol collectively constituted over 50% of total alcohols in the HM+CF and FTF+CF groups. Similarly, the contents of 8 esters and 9 ketones varied significantly. FTF supplementation notably increased ester and ketone levels, with Butyl 2-methylbutanoate and 2-Pentanone (M) accounted for 59% and 51% of the total esters and ketones, respectively. Aldehyde levels were also differentially affected, with butanal (M) representing only 6% of total aldehydes in the CF group but rising to 51% in the BL+CF group. Eight “other” compounds also showed significant inter-group differences. The relative abundance of the five major VOC classes summarized the compositional shift (Figures 8A–E). The CF group was characterized by the highest proportions of aldehydes (9.83%) and ketones (30.70%). The maximum ester content (28.27%) was observed in the FTF+CF group, while the BL+CF group contained the highest alcohol level (21.14%). The HM+CF group featured the greatest proportion of “other” compounds (23.59%).

#### 3.6.3 Identification of flavor markers

**Figure 9:**
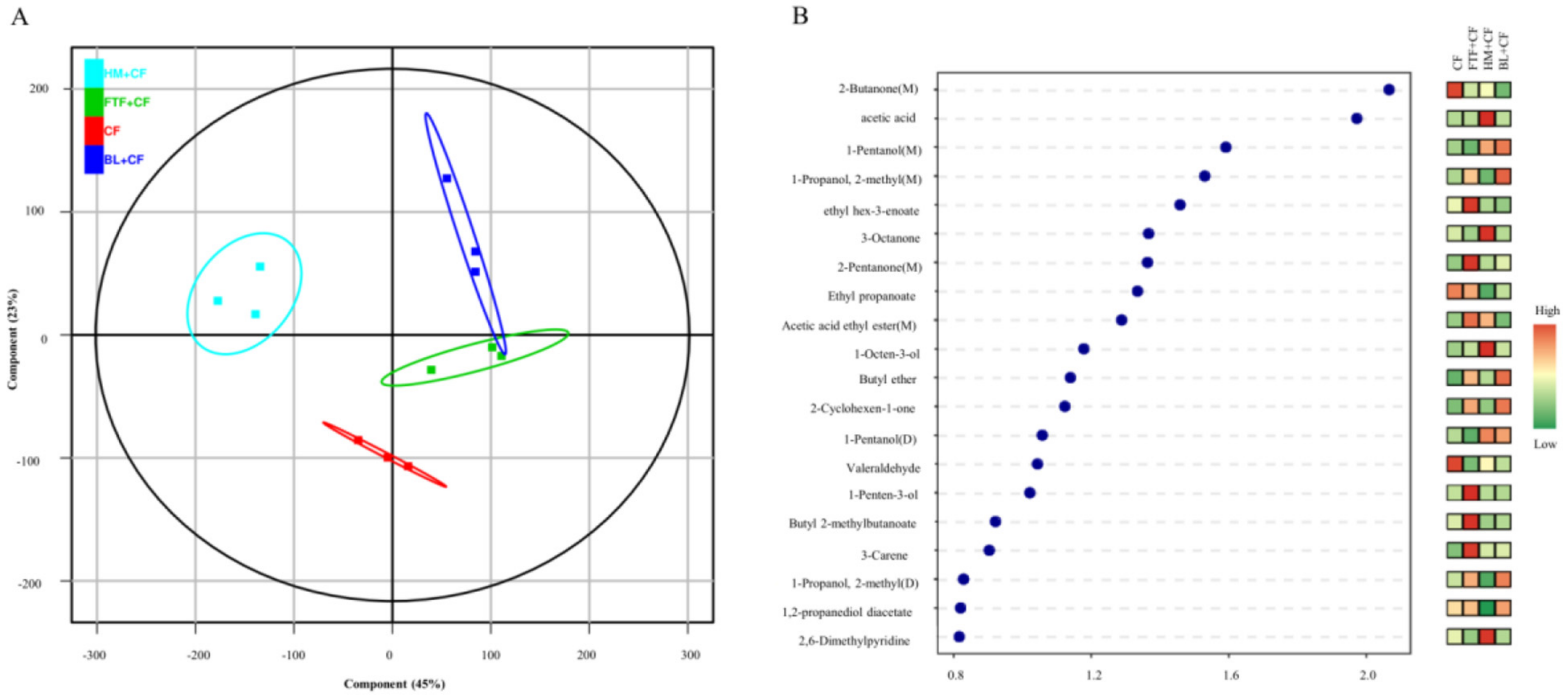
Proportions of VOCs (with flavor markers identified) in the four different experimental groups. **(A)** Plot of PLS-DA scores of VOCs; **(B)** Plot of VIP scores of VOCs.

The influence of various dietary additives on flavor profiles in *E. sinensis* was investigated by partial least squares-discriminant analysis (PLS-DA). The GC-IMS model demonstrated superior discriminatory efficacy compared to PCA, accounting for 68% of the total variance in flavor markers (Figure 9A). Based on variable importance in projection (VIP) scores, volatile compounds with values exceeding 1.0 were defined as significant flavor markers. Accordingly, 15 such volatiles were identified in *E. sinensis* (Figure 9B), categorized into five alcohols, four ketones, three esters, and three miscellaneous compounds. Notably, the 2-Butanone (M) yielded the highest VIP score.

### 3.7 Analysis of intestinal microbiota

#### 3.7.1 OTUs characterization and community structure of intestinal microbiota

**Figure 10:**
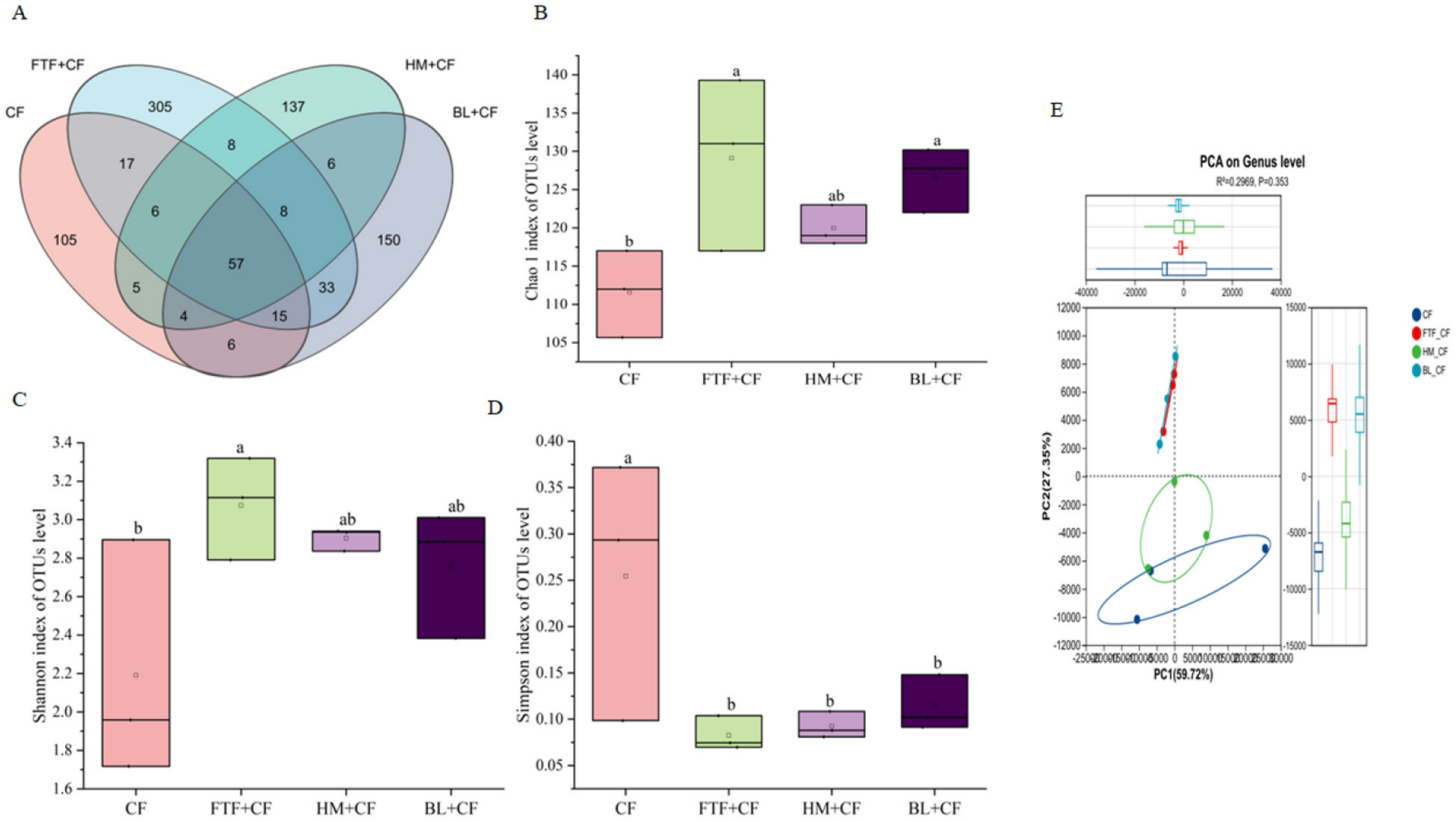
Intestinal microbial composition in experimental groups and the control group (*n* = 3). **(A)** represents the Venn diagram of different microorganisms **(B–D)** represent the biodiversity index of Chao, Simpson and Shannon in OUTs level, respectively; **(E)** represents Principal coordinate analysis (PCoA) plot based on Bray-Curtis distances showing the clustering of intestinal microbial communities across the four dietary groups. PERMANOVA (adonis) results: *R*^2^ = 0.3687, *P* = 0.084 (999 permutations). Different lowercase letters indicate significant differences between groups (*P* < 0.05).

High-throughput 16S rRNA sequencing was employed to characterize the intestinal bacterial communities of *E. sinensis*. Following quality control of raw reads, over 90% of the sequences were retained as high-quality data, which were subsequently clustered into 862 operational taxonomic units (OTUs). Venn diagram analysis indicated that 37 OTUs were common to all four experimental groups, while 105, 305, 137, and 150 OTUs were unique to the CF, FTF+CF, HM+CF, and BL+CF groups, respectively (Figure 10A). Analysis of cecal alpha diversity revealed a significantly higher Simpson index in the CF group compared to the other groups (*P* < 0.05). In contrast, the Chao 1 and Shannon indices were elevated in the experimental groups relative to the CF group (Figures 10B–D).

Principal coordinate analysis (PCoA) based on Bray-Curtis distances was performed to visualize beta diversity patterns among groups (Figure 10E). The PCoA plot showed that the microbial communities of the FTF+CF and BL+CF groups clustered together, with no clear separation between them. Similarly, the CF and HM+CF groups also formed a distinct cluster, showing minimal separation (Figure 10E). However, this visual separation was not statistically supported, as the initial analysis yielded a *P*-value of 0.353, indicating no significant difference among groups. To statistically evaluate the significance of these observed groupings, permutational multivariate analysis of variance (PERMANOVA, adonis) was conducted with 999 permutations. The PERMANOVA results indicated that the overall differences among the four dietary groups were not statistically significant (*R*^2^ = 0.3687, *P* = 0.084), suggesting that while visual clustering trends were observed, the microbial community structures across treatments did not reach statistical significance at the conventional *P* < 0.05 threshold.

#### 3.7.2 Analysis of bacterial communities' components

**Figure 11:**
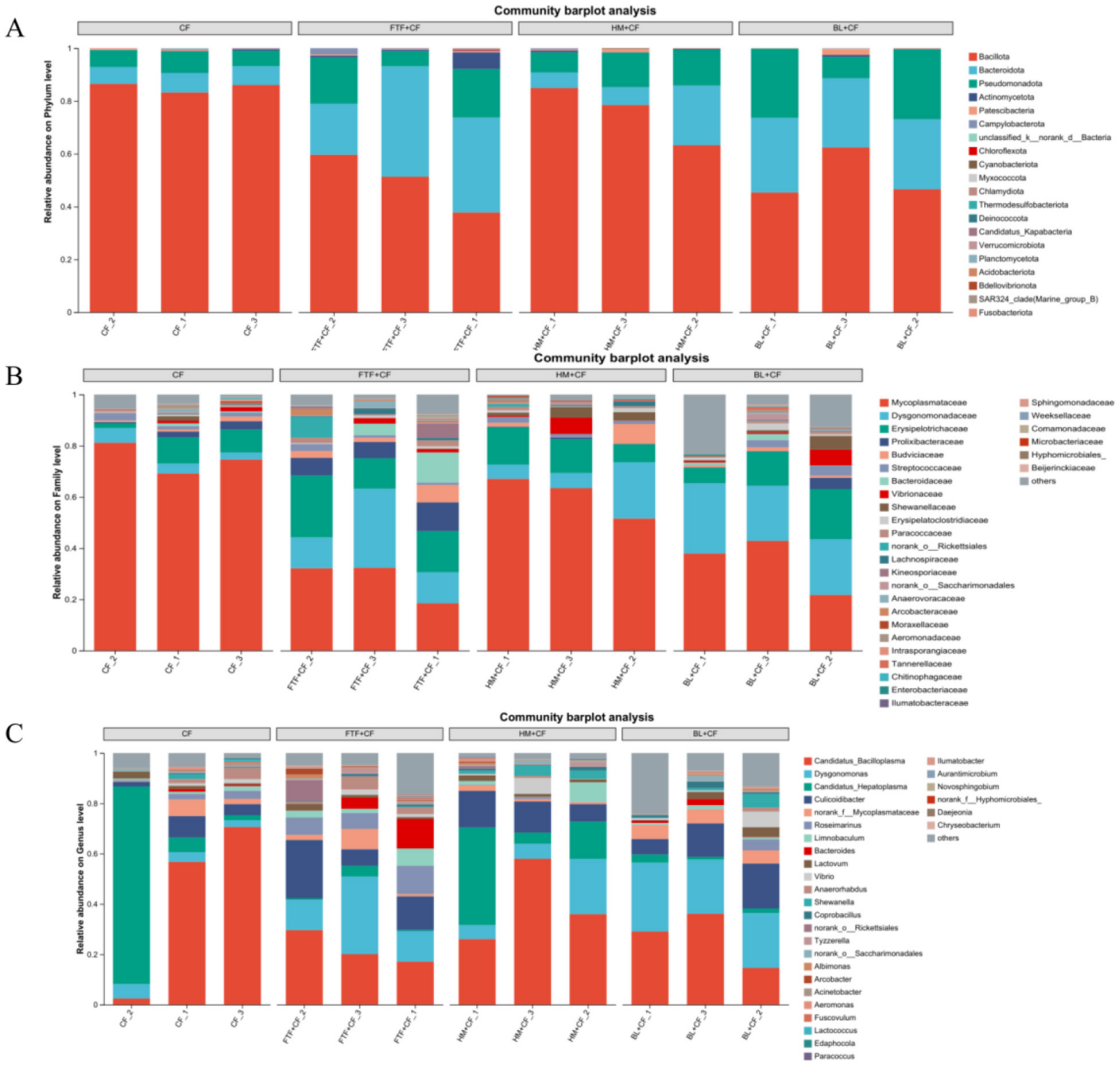
Alterations in microbiota at the phylum, family, and genus levels. **(A–C)** represent the relative abundance of OTUs in phylum, family and genus level, respectively.

Based on taxonomic abundance, the identified OTUs were classified into 20 phyla, 31 families, and 31 genera (Figure 11). Across the four experimental groups, three phyla—Bacillota, Bacteroidota, and Pseudomonadota—were consistently dominant (Figure 11A). At the family level, dietary supplementation with FTF, HM, or BL increased the relative abundance of Mycoplasmataceae, which constituted a major component of the cecal microbiota (Figure 11B). In contrast, the abundances of Dysgonomonadaceae and Erysipelotrichaceae were slightly lower in the CF group compared to the other three experimental groups. At the genus level, Candidatus_Bacilloplasma, Dysgonomonas, and Candidatus_Hepatoplasma were the three most abundant bacterial taxa shared among all groups (Figure 11C).

#### 3.7.3 Functional analysis of bacterial communities

**Figure 12:**
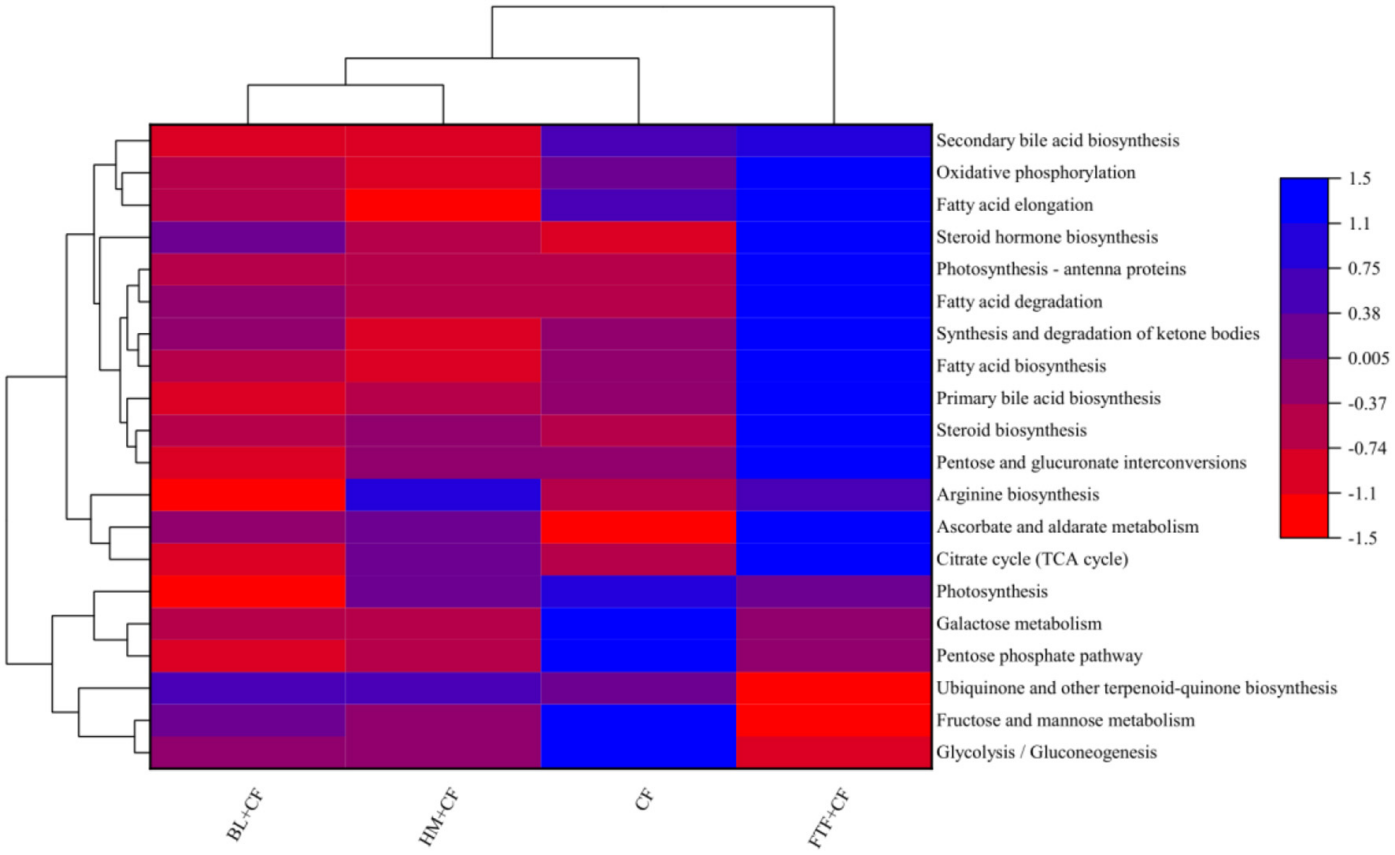
Predicted functions of the intestinal microbiota of *E sinensis* fed on CF, FTF, HM, and BL in KEGG level 3.

Tax4Fun was utilized to predict the functional profiles of the intestinal microbiota in *E. sinensis* fed different diets, based on the KEGG database. At KEGG level 1, metabolic functions dominated the microbial activities across all dietary groups. Specifically, in the CF group, 71.57% of the predicted functions were metabolism-related (Supplementary Table S3). These were further delineated at level 2 into carbohydrate metabolism (9.56%), amino acid metabolism (6.82%), energy metabolism (3.71%), and lipid metabolism (2.85%) (Figure 12). A similar pattern was observed in the other three groups (FTF + CF, HM + CF, and BL + CF), where metabolism also represented the primary functional category, accounting for 73.88%, 70.95%, and 69.95% of level 1 functions, respectively (Supplementary Table S4). At level 2, the predominant metabolic subcategories included carbohydrate metabolism (9.02%, 8.76%, 8.83%), amino acid metabolism (7.62%, 7.30%, 6.92%), energy metabolism (3.83%, 3.66%, 3.65%), and lipid metabolism (3.20%, 2.74%, 2.88%) for the FTF + CF, HM + CF, and BL + CF groups, respectively (Figure 12). In KEGG level 3, 20 pathways exhibited the most pronounced differences relative to the CF group (Figure 12). Compared with the CF group, the FTF+CF group showed significant enrichment in glycolysis/gluconeogenesis, fructose and mannose metabolism, and ubiquinone and other terpenoid-quinone biosynthesis. In contrast, the HM+CF and BL+CF groups were notably enriched in secondary bile acid biosynthesis and fatty acid biosynthesis. Conversely, the CF group itself demonstrated higher abundance in steroid biosynthesis and ascorbate and aldarate metabolism compared to the other three groups.

## 4 Discussion

The growth and development of crabs involve a complex interplay of internal and external factors, with diet being a crucial external determinant. The present study explored the potential of incorporating beef liver (BL), housefly maggot (HM), and frozen trash fish (FTF) as novel ingredients in aquafeeds for *E. sinensis*. Notably, substituting a portion of the commercial feed (CF) with FTF did not compromise growth performance, suggesting its viability as an alternative protein source without adverse effects. This finding aligns with previous studies in other aquatic species such as *Trachinotus ovatus* (34), *Epinephelus fuscoguttatu* × *Epinephelus lanceolatus* (72), and *Portunus trituberculatus* (35). In contrast, partial replacement of CF with BL or HM significantly enhanced multiple growth metrics. This growth-promoting effect is consistent with observations in rainbow trout fed HM-supplemented diets (36). Animal-based protein sources for feed typically contain a high concentration of digestible protein, energy-rich fats, and bioavailable minerals, which enable better to fulfill their nutritional requirements (87). Specifically for the BL group, the observed growth enhancement may be mechanistically linked to the presence of bioactive factors inherent in liver tissue, such as insulin-like growth factor 1 (*IGF-1*) and other growth-promoting peptides that are known to be abundant in animal liver and have been shown to stimulate protein synthesis and muscle cell proliferation in various species. This hypothesis warrants further investigation into the direct effects of dietary BL on the endocrine regulation of growth in crustaceans. This result indicates that different feed sources have a significant impact on the absorption and metabolism of nutrients in crabs.

The nutritional quality of aquaculture products is fundamentally reflected in their proximate composition (37). In this study, BL supplementation led to significantly higher crude protein and ash content in crab muscle compared to other groups (Figures 1G, I). This enhancement, coupled with the comprehensive amino acid enrichment discussed later, strongly suggests that BL not only provides a rich substrate but also actively promotes protein anabolism. As proposed above, the potential involvement of IGF-1 or similar growth factors in BL could facilitate protein digestion and absorption rates, leading to more efficient nitrogen retention and muscle protein accretion. Conversely, the FTF diet resulted in the highest crude fat deposition (Figure 1H), a phenomenon potentially linked to its high cholesterol content, which is known to influence lipid metabolism and deposition in crustaceans (38). Furthermore, the elevated PUFA content in FTF, particularly EPA and DHA, may modulate the activity or gene expression of key lipogenic enzymes (e.g., fatty acid synthase, elongases, and desaturases) in the hepatopancreas and muscle, as has been recently demonstrated in other aquatic species (39). This potential regulatory effect of dietary PUFAs on endogenous lipid metabolism could contribute to the distinct fatty acid deposition patterns observed, and warrants targeted molecular investigations in future studies.

The innate immune system, comprising cellular and humoral components, is paramount for pathogen defense in crustaceans (40). Within this system, hemocytes are central to cellular responses and the release of humoral factors (41). Concurrently, the antioxidant system, with key enzymes such as SOD and CAT, provides essential protection against oxidative stress induced by infection or environmental challenges (42, 43). The significant elevation of SOD, CAT, LZM, and AKP activities in crabs fed HM or FTF suggests a diet-induced enhancement of both antioxidant capacity and innate immune defense. This effect may be linked to the rich sterol and phospholipid content of these feed ingredients (44). Dietary lipids are crucial for crustaceans, not only as a source of energy and essential fatty acids but also as carriers of fat-soluble vitamins and as structural components that support membrane integrity and various metabolic functions, thereby potentially upregulating associated enzyme systems (45, 46). A notable and contrasting finding was the concurrent rise in MDA levels and suppression of ACP activity observed specifically in the HM group. MDA is a well-established biomarker of lipid peroxidation and oxidative damage (47), while ACP is a hydrolytic enzyme involved in pathogen clearance and immune regulation (48). This combination—elevated oxidative damage alongside diminished immune enzyme activity—indicates that HM supplementation, while boosting certain antioxidant pathways, may have induced a state of peroxidative stress and partially compromised specific aspects of the humoral immune response in *E. sinensis*. The precise nutritional components in HM responsible for this divergent effect require further elucidation.

According to Jobling (49), several fatty acids—C18:2n-6 (linoleic acid, LA), C18:3n-3 (α-linolenic acid, LNA), C20:5n-3 (EPA), and C22:6n-3 (DHA)—are considered essential for crustaceans. In the present study, analysis of fatty acid composition revealed that crabs in the FTF+CF group exhibited significantly higher levels of these essential fatty acids (EFAs) in muscle tissue compared to other groups. Frozen fresh fish, a natural feed, is known to be rich in fatty acids and proteins. Supporting this, Wang et al. (35) reported that dietary supplementation with frozen fresh fish enhances highly unsaturated fatty acid content in the hepatopancreas and gonads of the three-spotted swimming crab, which aligns with our findings. While freshwater crustaceans may achieve adequate growth with dietary n-6 polyunsaturated fatty acids (PUFAs) alone (49), supplementation with n-3 highly unsaturated fatty acids (HUFAs) has been recommended to delay early maturation (50). Additionally, both n-6 and n-3 PUFAs are essential for animals' health, contributing to lipid metabolism regulation and prevention of hypertension. These fatty acids compete in shared biosynthetic and metabolic pathways; thus, elevated n-3 PUFA levels often result in reduced n-6 PUFA content (51), a pattern also observed in our study. Higher intake of n-3 PUFAs facilitates the synthesis of n-3 lipoxins, which are beneficial to human health. This may explain the lower ∑n-6 PUFA content in the FTF group compared to the BL+CF group. However, previous studies have indicated that the fatty acid content of *L. calcarifer* (52) or R. (*L*.) *catesbeiana* (19) are not significantly affected by diets supplemented with HM. This conclusion aligns with our current study that no distinct changes also observed for the fatty acid composition of crab muscle in group HM+CF. Collectively, these findings suggest that FTF, owing to its direct provision of preformed HUFAs and potential regulatory effects on lipid metabolic enzymes (as discussed above), represents an optimal dietary source for enhancing the nutritional lipid profile of farmed *E. sinensis*.

The nutritional value of aquatic products as a protein source is largely determined by their amino acid composition, which is influenced by factors such as diet, genetics, and environment (37). In this study, dietary supplementation with beef liver (BL) profoundly reshaped the muscle amino acid profile of *E. sinensis*, leading to a comprehensive enrichment of both essential and non-essential amino acids. This broad enhancement suggests that BL not only provides a rich and balanced amino acid substrate but may also improve the overall efficiency of protein synthesis or retention within muscle tissue. As hypothesized earlier regarding growth performance, the presence of growth-promoting factors in BL, such as *IGF-1*, could directly stimulate the protein synthesis machinery, enhancing the uptake and incorporation of dietary amino acids into muscle. This mechanism would explain the concurrent increases in growth, crude protein, and nearly all individual amino acids observed exclusively in the BL group, distinguishing it from the more selective effects seen with other dietary treatments.

The flavor profile of crab is closely associated with its delicious amino acid (DAA) content, particularly aspartic acid (Asp), glutamic acid (Glu), glycine (Gly), alanine (Ala), serine (Ser), and threonine (Thr), which contribute to umami and sweet tastes. The BL+CF group showed a marked increase in total DAA levels, which directly correlates with the observed sensory superiority. Furthermore, the significant elevation of proline (Pro) in this group is of particular interest. As proline is implicated in enhancing energy metabolism and supporting muscle protein synthesis (53), its increased deposition may provide a metabolic basis for the concurrent improvement in growth performance observed in BL-fed crabs, a phenomenon supported by similar findings in *Nile tilapia* (54).

Valine (Val), an essential amino acid for crustaceans, serves as a multifunctional molecule involved in protein anabolism (55), energy production (56), and metabolic regulation (57). A key finding from the nutritional assessment was the consistent identification of Val as the first limiting amino acid across all diets, regardless of their formulation. This indicates that the muscle tissue of *E. sinensis* has a particularly high requirement for Val within its r formulation. This indicates that the muscle tissue of anabolism BL-fed crabs, a phenomeu), glycine (Gly), alanine (Ala), serine (Ser), and threonine (Thr), which contribute to umami and sweeetary supply, suggesting Val should be a primary target in future feed optimization for this species.

The EAA/TAA ratio across all groups showed minimal variation, averaging 0.41, a value marginally higher than the FAO/WHO standard of 0.4. This slight elevation may be linked to the expansive (20,000 m^2^) culture ponds used in this study, which provided a conducive environment for increased swimming activity. Enhanced swimming intensity has been previously associated with improved amino acid deposition, as evidenced in species like largemouth bass under circulating water conditions (58). Consequently, the results demonstrate that dietary BL supplementation can enhance the amino acid profile of crabs, particularly enriching those compounds associated with superior freshness and flavor.

Minerals are essential micronutrients that must be obtained exogenously, playing critical roles in osmoregulation, enzyme function, and structural integrity in aquatic animals (59). The distinct mineral profiles observed in this study directly reflect the elemental composition of the respective feed ingredients. The significantly elevated levels of K, Fe, and Cu in the BL+CF group can be attributed to the well-documented richness of beef liver in these bioavailable minerals. This effective transfer from diet to tissue underscores the potential of BL as a superior source for replenishing key trace elements in aquaculture feeds, which may support enhanced enzymatic and metabolic functions in crabs (27, 28).

Conversely, the highest Na deposition in the FTF+CF group likely originates from the marine environment of the anchovy-based feed. The elevated Ca content in this group may be linked to a dual mechanism: the direct dietary input from fishbone and a potential physiological adaptation where culturing in higher alkalinity environments stimulates muscle fiber development and associated mineral deposition, as suggested by studies in other species (60). The consistently low mineral levels in the HM+CF and CF groups highlight the limitations of these diets in supplying critical inorganic nutrients. This comparative deficiency may represent a nutritional constraint that could affect long-term physiological resilience and product quality, emphasizing the importance of mineral-specific supplementation in formulated feeds for optimal crustacean nutrition.

The flavor of crab is profoundly affected by aldehydes. Whereas, unsaturated aldehydes contribute grassy or fruity aromas, their saturated counterparts are recognized as principal determinants of the earthy odor in aquatic organisms (61). Our analysis identified four saturated aldehydes, among which heptanal and butanal (M) were present at peak levels in the BL+CF group, which also had the highest total aldehyde proportion. These sensory data suggest that BL supplementation enhances the earthy odor of *E. sinensis* muscle. To elucidate the origin of these aldehydes, we consider the well-established pathway of lipid oxidation, wherein PUFAs serve as major precursors (62). The BL+CF group contained significantly higher levels of PUFAs compared to other groups, providing a plausible substrate basis for increased aldehyde formation. This aligns with the proposed mechanistic sequence: dietary BL, rich in PUFAs, leads to enhanced PUFA deposition in muscle, which subsequently serves as a substrate for lipid oxidation during storage or processing, generating characteristic volatile aldehydes such as butanal and heptanal. This “feed components—nutrient deposition—precursor substances—flavor compoundsncesher groups, providing a plausible substrate basis for increased aldehyde formatio.

In this study, alcohols were the most frequently identified volatile compounds, typically generated through lipolysis. These compounds contribute pleasant aromas, including fruity and floral notes. Compared to short straight-chain alcohols, longer straight-chain or branched alcohols exhibit lower flavor thresholds and exert a more pronounced influence on the flavor profile of aquatic animal muscle (63, 64). For instance, 1-pentanol imparts a grassy note, while 2-butanol offers fruity and creamy nuances (37). The CF group showed the lowest total alcohol content (15.96%, Figure 8A), whereas the BL+CF group exhibited the highest proportion (21.14%, Figure 8B). These findings suggest that dietary supplementation with animal protein sources significantly affects the muscle quality of *E. sinensis*, particularly in terms of flavor attributes. Additionally, alcohols serve as precursors for ester synthesis via esterification. Esters such as propanoates emit sweet fruity scents, and ethyl propanoate conveys a pineapple-like aroma (65). In the present study, the proportion of esters was higher in the FTF+CF groups than in the CF group. The elevated ester content in the FTF group warrants further investigation into its origins. Esters are typically formed through the esterification of alcohols and fatty acids, a process that can be mediated by microbial metabolism within the gut or by endogenous enzymes in the muscle tissue. Given that the FTF diet significantly modulated the gut microbiota composition (as discussed below), it is plausible that specific microbial populations enriched by FTF possess enhanced ester-synthesizing capabilities. Future studies should employ metagenomic or metabolomic approaches to identify specific microbial taxa (e.g., certain bacteria within the Bacteroidota phylum) and their functional genes (e.g., esterases, alcohol acyltransferases) that may contribute to the characteristic ester profile observed in the FTF group. Furthermore, elevated aldehyde levels in muscle tissue can lead to undesirable off-flavors (18). Our results indicated that crabs fed exclusively commercial feed contained the highest aldehyde content (9.83%). In summary, dietary supplementation with FTF, BL, or HM effectively reduced the concentration of undesirable aldehydes while enhancing the levels of favorable flavor compounds such as alcohols and esters. This shift mitigated the earthy odor of crab and enhanced appealing aromas such as fruity and milky notes, which are preferred by consumers.

Dietary composition is a key determinant of gut microbiota structure, which in turn critically influences host intestinal health. In crustaceans, feed formulations have been shown to selectively modulate intestinal microbial communities (66). Dietary composition is a key determinant of gut microbiota structure, which in turn critically influences host intestinal health. In crustaceans, feed formulations have been shown to selectively modulate intestinal microbial communities (25), and other crustaceans, including *Scylla paramamosain* (67), *Callinectes sapidus* (68) and *Macrobrachium rosenbergii* (69). Furthermore, dietary regimens involving CF and HM+CF led to an increased relative abundance of Bacillota in *E. sinensis*. This phylum encompasses several probiotic genera—such as Lactobacillus, Enterococcus, and Bacillus (70) —known to inhibit pathogens, improve digestive and immune functions, and support intestinal homeostasis in aquatic organisms (71). The enrichment of Bacillota in the HM+CF group may therefore contribute to improved intestinal health, a pattern analogous to that observed in juvenile hybrid grouper fed formulated diets (72).

Additionally, Bacteroidota was identified as another predominant phylum in E. sinensis, with its relative abundance elevated under the FTF+CF and BL+CF feeding regimes. This phylum is recognized as a common dominant microbial component in crustaceans (73) and is implicated in nutrient cycling and organic matter mineralization (69). Although Ding et al. suggested that an increased relative abundance of Bacteroidota may be associated with impaired growth performance in Procambarus clarkii (74), the functional role of this phylum in crabs remains poorly understood.

Functional prediction analysis (Tax4Fun) was employed to explore potential functional differences in the intestinal microbiota across dietary groups (Figure 12). The analysis suggested that all groups exhibited a conserved metabolic potential, predominantly enriched in core pathways such as carbohydrate and amino acid metabolism, consistent with reported microbial metabolic traits in crustaceans (25, 75, 76). Notably, while studies in fish have linked formulated diets to enhanced pyrimidine and nucleotide sugar metabolism (72), the CF group in this study displayed distinctive enrichment in steroid biosynthesis and ascorbate-aldarate metabolism. Given that steroid biosynthesis represents a terminal synthetic step strongly dependent on glycolytic carbon and reducing equivalents (77), these observations may suggest a potential mechanistic link: dietary protein source substitution could potentially reshape microbial metabolic routing, preferentially channeling glycolytic flux toward specialized biosynthetic pathways. This functional shift might imply that nutrient composition modulates microbial metabolism not merely quantitatively, but also qualitatively, redirecting core carbon flow toward specific secondary pathways. However, it is important to acknowledge the methodological limitations of Tax4Fun for functional prediction from 16S rRNA data. Tax4Fun infers KEGG pathway abundances based on sequence similarity to reference genomes, and its accuracy depends on the availability of closely related genomes in the database. Given that reference genomes for crustacean gut microbiota are not yet well-established, these functional predictions should be interpreted with caution as preliminary indications of potential functional differences rather than definitive conclusions.

Integrating gut microbial composition with host phenotypic outcomes reveals coherent patterns suggesting diet-microbiota-host interactions. The clustering of microbial communities, with FTF+CF and BL+CF groups forming one cluster distinct from CF and HM+CF (Figure 10E), paralleled patterns in fatty acid composition (Figure 3A), indicating a fundamental link between microbial community structure and host lipid metabolism.

The FTF+CF group exhibited the most distinctive profile, with elevated Bacteroidota abundance (Figure 11A) and enrichment of glycolysis-related pathways (Figure 12), coinciding with the highest muscle PUFA (EPA/DHA) levels (Figure 3B), highest ester content (28.27%, Figure 7D), and elevated Na deposition (Figure 5E). The parallel enrichment of Bacteroidota—a phylum implicated in lipid metabolism (69)—with enhanced PUFA deposition and ester generation suggests this phylum may modulate both host lipid profiles and flavor compound formation.

The BL+CF group, while clustering with FTF+CF in microbial structure (Figure 10E) and fatty acid profiles (Figure 3A), displayed a distinct phenotypic signature: highest crude protein and ash contents (Figures 1G, I), comprehensive amino acid enrichment (17 of 18 amino acids elevated, Supplementary Table S2), highest K, Fe, and Cu levels (Figures 5A, C, E), and greatest alcohol content (21.14%, Figure 7C). Enrichment of fatty acid biosynthesis pathways in the BL+CF group (Figure 12) aligned with elevated muscle crude fat (Figure 1H), suggesting microbial lipid metabolism may contribute to host fatty acid accumulation.

The HM+CF group, clustering with CF in microbial composition (Figure 10E), showed minimal differences from control in proximate composition and fatty acid profiles, but exhibited the most dramatic shift in volatile compounds, with prominent red signals in differential topographic mapping (Figure 6B) and the highest proportion of “other” compounds (23.59%, Figure 8E), including unique elevation of 2,3,5-trimethylpyrazine (Figure 7A). Notably, this group displayed a paradoxical response: while SOD, CAT, LZM, and AKP were elevated (Figures 2A, C–E), it also exhibited the highest MDA and lowest ACP activity (Figures 2B, F), suggesting HM supplementation may induce oxidative stress while enhancing certain antioxidant pathways—a pattern potentially linked to its unique VOC profile.

The CF group, characterized by the highest Simpson index but lower diversity metrics in experimental groups (Section 3.7.1), showed distinctive enrichment in steroid biosynthesis (Figure 12), highest aldehyde content (9.83%, Figure 7G), and lowest alcohol content (15.96%, Figure 8B). The unique functional pathways enriched in the CF group may reflect microbial metabolic adaptation to formulated feed distinct from animal-derived protein supplements.

These parallel observations across experimental groups provide compelling evidence for linkages between dietary intervention, gut microbial community structure, and host phenotypic outcomes. The coherence between microbial shifts and host physiological responses—evident in consistent clustering patterns, group-specific enrichment of microbial taxa and functional pathways, and their correspondence with distinctive nutritional and flavor profiles—supports the hypothesis that gut microbiota mediates, at least in part, the effects of diet on crab quality traits. This interpretation aligns with the proposed framework of “feed components—microbial metabolism—nutrient deposition/flavor precursor generation—host quality traits.

Collectively, these findings illustrate that dietary interventions not only alter the taxonomic profile of the gut microbiota in *E. sinensis* but also modulate its functional landscape. However, the functional plasticity of crustacean gut microbiota remains underexplored, warranting further mechanistic studies to elucidate how dietary components reprogram microbial metabolic networks and ultimately influence host health and physiology.

## 4 Conclusion

This study demonstrates that dietary supplementation with BL, FTF, and HM distinctly modulates the nutritional composition, volatile flavor profile, and underlying physiology of *E. sinensis*, with BL and FTF emerging as the most promising strategies for quality improvement. BL is particularly effective for enhancing fundamental nutritional quality and umami flavor, significantly increasing crude protein, essential and delicious amino acids, and key minerals (K, Fe, Cu). In contrast, FTF is ideally suited for enriching health-beneficial PUFAs, notably EPA and DHA, while simultaneously improving the volatile profile by reducing earthy odors and enhancing fruity and milky notes. These quality improvements were supported by enhanced antioxidant (SOD, CAT) and immune-related (LZM, AKP) enzyme activities, as well as modulated gut microbiota and enriched metabolic pathways related to lipid and amino acid metabolism, providing a mechanistic link between diet, physiology, and product quality. However, this study has limitations, including the absence of long-term validation across multiple culture cycles and lack of cost-effectiveness analysis, which should be addressed in future research to assess practical feasibility for commercial aquaculture. Collectively, these findings offer a valuable reference for assessing the economic efficacy of alternative animal protein ingredients in *E. sinensis* aquaculture.

## Data Availability

The data presented in this study are publicly available. The data can be found here: https://www.ncbi.nlm.nih.gov/, accession number PRJNA1444304.
