# Supplementary material for: Linking diet to growth, nutrient composition, and flavor characteristics in Chinese mitten crab (Eriocheir sinensis): a study based on biochemical composition and intestinal microbiota
Source: Front Nutr. 2026 Apr 1;13:1798709. doi: 10.3389/fnut.2026.1798709 (PMC13082253; doi:10.3389/fnut.2026.1798709)
Supplement: Supplementary file 1 [file Table_1.docx]

Table S1 Fatty acid composition and content in muscle of Chinese mitten crab (g/100g)

| Fatty acid | CF | BL+CF | HM+CF | FTF+CF | Source | df | Mean Square | Sig. |
| --- | --- | --- | --- | --- | --- | --- | --- | --- |
| C14:0 | NA | NA | NA | 0.014±0.004 | Treatment | 3 | 2.1×10^-4^ | 1.6×10^-4^ |
|  |  |  |  |  | Error | 12 | 1.3×10^-5^ |  |
| C15:0 | NA | NA | NA | 0.004±0.0003 | Treatment | 3 | 1.6×10^-5^ | <0.0001 |
|  |  |  |  |  | Error | 12 | 6.7×10^-8^ |  |
| C16:0 | 0.076±0.005^c^ | 0.012±0.01^ab^ | 0.084±0.002^bc^ | 0.19±0.02^a^ | Treatment | 3 | 0.01032 | 1.1×10^-4^ |
|  |  |  |  |  | Error | 12 | 5.9×10^-4^ |  |
| C16:1 | 0.013±0.003^b^ | 0.032±0.008^b^ | 0.016±0.0003^b^ | 0.055±0.01^a^ | Treatment | 3 | 0.0015 | 0.00689 |
|  |  |  |  |  | Error | 12 | 2.3×10^-4^ |  |
| C17:0 | NA | NA | NA | 0.0075±0.0002 | Treatment | 3 | 5.7×10^-5^ | <0.0001 |
|  |  |  |  |  | Error | 12 | 3.4×10^-8^ |  |
| C18:0 | 0.066±0.002^b^ | 0.013±0.005^c^ | 0.065±0.002^b^ | 0.0894±0.006^a^ | Treatment | 3 | 0.00325 | <0.0001 |
|  |  |  |  |  | Error | 12 | 7.2×10^-5^ |  |
| C18:1n9c | 0.11±0.007^c^ | 0.16±0.01^b^ | 0.12±0.003^c^ | 0.21±0.01^a^ | Treatment | 3 | 0.00727 | <0.0001 |
|  |  |  |  |  | Error | 12 | 2.9×10^-4^ |  |
| C18:2n6c (LA) | 0.052±0.004^c^ | 0.085±0.01^a^ | 0.073±0.002^ab^ | 0.059±0.006^bc^ | Treatment | 3 | 8.5×10^-4^ | 0.01301 |
|  |  |  |  |  | Error | 12 | 1.6×10^-4^ |  |
| C20:0 | 0.0095±0.0002^ab^ | 0.010±0.0005^ab^ | 0.0086±0.0004^bc^ | 0.011±0.001^a^ | Treatment | 3 | 0.0081 | 0.0081 |
|  |  |  |  |  | Error | 12 | 0.0111 |  |
| C18:3n3 (LNA) | 0.0079±0.001^ab^ | 0.010±0.001^ab^ | 0.011±0.001^a^ | 0.0075±0.001^bc^ | Treatment | 3 | 1.4×10^-5^ | 0.11545 |
|  |  |  |  |  | Error | 12 | 5.6×10^-6^ |  |
| C20:1 | 0.0056±0.0005^b^ | 0.043±0.0002^b^ | 0.0048±0.0001^b^ | 0.013±0.001^a^ | Treatment | 3 | 6.4×10^-5^ | <0.0001 |
|  |  |  |  |  | Error | 12 | 1.5×10^-6^ |  |
| C20:2 | 0.014±0.0005^a^ | 0.012±0.0005^b^ | 0.010±0.0003^b^ | 0.011±0.001^b^ | Treatment | 3 | 8.8×10^-6^ | 0.02325 |
|  |  |  |  |  | Error | 12 | 1.9×10^-6^ |  |
| C22:0 | 0.011±0.0004 | 0.011±0.0007 | 0.0082±0.0001 | 0.011±0.001 | Treatment | 3 | 5.7×10^-6^ | 0.17831 |
|  |  |  |  |  | Error | 12 | 2.9×10^-6^ |  |
| C20:3n6 | NA | 0.011±0.002 | NA | NA | Treatment | 3 | 1.2×10^-4^ | <0.0001 |
|  |  |  |  |  | Error | 12 | 2.5×10^-6^ |  |
| C22:1n9 | 0.0056±0.0002^ab^ | 0.0041±0.0004^b^ | 0.0051±0.0007^ab^ | 0.0062±0.0007^a^ | Treatment | 3 | 3.1×10^-6^ | 0.09885 |
|  |  |  |  |  | Error | 12 | 1.2×10^-6^ |  |
| C20:4n6 (ARA) | 0.033±0.0008^b^ | 0.093±0.003^a^ | 0.025±0.0004^c^ | 0.031±0.002^b^ | Treatment | 3 | 0.004 | <0.0001 |
|  |  |  |  |  | Error | 12 | 1.1×10^-5^ |  |
| C20:5n3 (EPA) | 0.072±0.002^b^ | 0.055±0.004^c^ | 0.065±0.003^bc^ | 0.089±0.008^a^ | Treatment | 3 | 7.9×10^-4^ | 0.00194 |
|  |  |  |  |  | Error | 12 | 8.7×10^-5^ |  |
| C24:1 | NA | NA | NA | 0.0040±0.0002 | Treatment | 3 | 1.6×10^-5^ | <0.0001 |
|  |  |  |  |  | Error | 12 | 5.2×10^-8^ |  |
| C22:6n3 (DHA) | 0.066±0.004^b^ | 0.048±0.00^c^ | 0.059±0.003^bc^ | 0.11±0.007^a^ | Treatment | 3 | 0.00294 | <0.0001 |
|  |  |  |  |  | Error | 12 | 8.5×10^-5^ |  |
| Σn-3 | 0.15±0.007^b^ | 0.11±0.005^c^ | 0.14±0.006^bc^ | 0.21±0.01^a^ | Treatment | 3 | 0.02504 | <0.0001 |
|  |  |  |  |  | Error | 12 | 7.7×10^-4^ |  |
| Σn-6 | 0.086±0.005^b^ | 0.19±0.01^a^ | 0.10±0.002^b^ | 0.090±0.007^b^ | Treatment | 3 | 0.01822 | <0.0001 |
|  |  |  |  |  | Error | 12 | 0.001 |  |
| Σn-3/n-6 | 1.72±0.07^b^ | 0.61±0.06^d^ | 1.37±0.05^c^ | 2.89±0.1^a^ | Treatment | 3 | 0.00566 | 0.00248 |
|  |  |  |  |  | Error | 12 | 6.5×10^-4^ |  |
| ∑SFA | 0.16±0.007^c^ | 0.26±0.02^b^ | 0.17±0.004^c^ | 0.32±0.02^a^ | Treatment | 3 | 0.0063 | <0.0001 |
|  |  |  |  |  | Error | 12 | 3.1×10^-4^ |  |
| ∑MUFA | 0.13±0.001^c^ | 0.20±0.02^b^ | 0.15±0.004^bc^ | 0.28±0.02^a^ | Treatment | 3 | 0.00958 | <0.0001 |
|  |  |  |  |  | Error | 12 | 2.2×10^-4^ |  |
| ∑PUFA | 0.25±0.01^b^ | 0.31±0.008^a^ | 0.24±0.008^b^ | 0.31±0.02^a^ | Treatment | 3 | 1.9708 | <0.0001 |
|  |  |  |  |  | Error | 12 | 0.02444 |  |

Note: Data were expressed as mean ± SD from four groups. Different superscript letters indicate significant differences (*P* < 0.05). Abbreviations: ∑MUFA, monounsaturated fatty acid; ∑PUFA, polyunsaturated fatty acid; ∑SFA, saturated fatty acid.
