# Supplementary material for: Linking diet to growth, nutrient composition, and flavor characteristics in Chinese mitten crab (Eriocheir sinensis): a study based on biochemical composition and intestinal microbiota
Source: Front Nutr. 2026 Apr 1;13:1798709. doi: 10.3389/fnut.2026.1798709 (PMC13082253; doi:10.3389/fnut.2026.1798709)
Supplement: Supplementary file 2 [file Table_2.docx]

| Amino acid | CF | BL+CF | HM+CF | FTF+CF | Source | df | Mean Square | Sig. |
| --- | --- | --- | --- | --- | --- | --- | --- | --- |
| Asp | 1.40±0.02^c^ | 1.58±0.009^a^ | 1.28±0.01^d^ | 1.44±0.04^b^ | Treatment | 3 | 0.05856 | <0.0001 |
|  |  |  |  |  | Error | 12 | 8.2×10^-4^ |  |
| Thr | 0.66±0.004^c^ | 0.71±0.003^a^ | 0.60±0.008^d^ | 0.68±0.005^b^ | Treatment | 3 | 0.00866 | <0.0001 |
|  |  |  |  |  | Error | 12 | 8.5×10^-5^ |  |
| Ser | 0.57±0.009^b^ | 0.61±0.01^a^ | 0.49±0.01^c^ | 0.57±0.006^b^ | Treatment | 3 | 0.01037 | <0.0001 |
|  |  |  |  |  | Error | 12 | 4.2×10^-4^ |  |
| Glu | 2.10±0.02^b^ | 2.28±0.01^a^ | 1.89±0.01^d^ | 2.05±0.03^c^ | Treatment | 3 | 0.10278 | <0.0001 |
|  |  |  |  |  | Error | 12 | 8.5×10^-4^ |  |
| Gly | 0.72±0.006^b^ | 0.83±0.02^a^ | 0.60±0.01^c^ | 0.74±0.04^b^ | Treatment | 3 | 0.03583 | <0.0001 |
|  |  |  |  |  | Error | 12 | 7.8×10^-4^ |  |
| Ala | 1.01±0.02^b^ | 1.09±0.03^a^ | 0.94±0.02^c^ | 1.05±0.006^ab^ | Treatment | 3 | 0.01587 | 0.00212 |
|  |  |  |  |  | Error | 12 | 0.00176 |  |
| Cys | 0.059±0.002^b^ | 0.03±0.001^a^ | 0.03±0.003^a^ | 0.030±0.003^a^ | Treatment | 3 | 7.9×10^-4^ | <0.0001 |
|  |  |  |  |  | Error | 12 | 1.9×10^-4^ |  |
| Trp | 0.17±0.001^c^ | 0.22±0.008^a^ | 0.18±0.004^b^ | 0.18±0.008^b^ | Treatment | 3 | 0.00188 | <0.0001 |
|  |  |  |  |  | Error | 12 | 9.6×10^-5^ |  |
| Val | 0.70±0.008^c^ | 0.82±0.006^a^ | 0.67±0.005^d^ | 0.74±0.02^b^ | Treatment | 3 | 0.01857 | <0.0001 |
|  |  |  |  |  | Error | 12 | 2.0×10^-4^ |  |
| Met | 0.15±0.009^c^ | 0.22±0.005^b^ | 0.20±0.01^b^ | 0.34±0.02^a^ | Treatment | 3 | 0.02444 | <0.0001 |
|  |  |  |  |  | Error | 12 | 3.7×10^-4^ |  |
| IIe | 0.64±0.008^b^ | 0.72±0.006^a^ | 0.60±0.006^c^ | 0.65±0.02^b^ | Treatment | 3 | 0.00996 | <0.0001 |
|  |  |  |  |  | Error | 12 | 2.2×10^-4^ |  |
| Leu | 1.13±0.003^c^ | 1.25±0.01^a^ | 1.04±0.009^d^ | 1.15±0.02^b^ | Treatment | 3 | 0.03137 | <0.0001 |
|  |  |  |  |  | Error | 12 | 2.9×10^-4^ |  |
| Tyr | 0.54±0.004^b^ | 0.59±0.008^a^ | 0.48±0.005^c^ | 0.59±0.006^a^ | Treatment | 3 | 0.01025 | <0.0001 |
|  |  |  |  |  | Error | 12 | 1.0×10^-4^ |  |
| Phe | 0.66±0.005^c^ | 0.74±0.01^a^ | 0.61±0.006^d^ | 0.70±0.01^b^ | Treatment | 3 | 0.01221 | <0.0001 |
|  |  |  |  |  | Error | 12 | 2.3×10^-4^ |  |
| Lys | 1.18±0.004^b^ | 1.27±0.02^a^ | 1.08±0.006^c^ | 1.18±0.01^b^ | Treatment | 3 | 0.02492 | <0.0001 |
|  |  |  |  |  | Error | 12 | 3.0×10^-4^ |  |
| His | 0.34±0.006^c^ | 0.41±0.01^a^ | 0.33±0.003^c^ | 0.37±0.03^b^ | Treatment | 3 | 0.00514 | <0.0001 |
|  |  |  |  |  | Error | 12 | 2.0×10^-4^ |  |
| Arg | 1.37±0.03^c^ | 1.72±0.01^a^ | 1.28±0.03^d^ | 1.46±0.01^b^ | Treatment | 3 | 0.14352 | <0.0001 |
|  |  |  |  |  | Error | 12 | 0.00193 |  |
| Pro | 0.63±0.009^c^ | 0.71±0.008^a^ | 0.63±0.007^c^ | 0.67±0.03^b^ | Treatment | 3 | 0.00621 | <0.0001 |
|  |  |  |  |  | Error | 12 | 4.1×10^-4^ |  |
| EAA | 5.61±0.02^c^ | 6.35±0.04^a^ | 5.30±0.04^d^ | 5.99±0.08^b^ | Treatment | 3 | 0.00188 | <0.0001 |
|  |  |  |  |  | Error | 12 | 9.6×10^-5^ |  |
| DAA | 6.45±0.02^b^ | 7.09±0.03^a^ | 5.80±0.07^c^ | 6.52±0.11^b^ | Treatment | 3 | 0.8345 | <0.0001 |
|  |  |  |  |  | Error | 12 | 0.00481 |  |
| TAA | 14.01±0.06^c^ | 15.79±0.08^a^ | 12.92±0.11^d^ | 14.52±0.19^b^ | Treatment | 3 | 5.72063 | <0.0001 |
|  |  |  |  |  | Error | 12 | 0.03154 |  |

Table S2 Amino acid composition and content of muscle in Chinese mitten crab (g/100g)
