# Supplementary material for: Linking diet to growth, nutrient composition, and flavor characteristics in Chinese mitten crab (Eriocheir sinensis): a study based on biochemical composition and intestinal microbiota
Source: Front Nutr. 2026 Apr 1;13:1798709. doi: 10.3389/fnut.2026.1798709 (PMC13082253; doi:10.3389/fnut.2026.1798709)
Supplement: Supplementary file 3 [file Table_3.docx]

Table S3 Predicted functions of the intestinal microbiota of *E sinensis* fed on CF, FTF, HM and BL in KEGG level 1.

| Pathway level1 | BL+CF | CF | FTF+CF | HM+CF |
| --- | --- | --- | --- | --- |
| Cellular Processes | 0.089455094 | 0.077480505 | 0.070798176 | 0.083875759 |
| Environmental Information Processing | 0.119887292 | 0.117334221 | 0.104754183 | 0.115387658 |
| Genetic Information Processing | 0.039862284 | 0.041646058 | 0.035954237 | 0.041639321 |
| Human Diseases | 0.03684423 | 0.033835662 | 0.035573707 | 0.035212252 |
| Metabolism | 0.699546067 | 0.715748382 | 0.738816655 | 0.709542339 |
| Organismal Systems | 0.014405033 | 0.013955172 | 0.014103041 | 0.014342671 |
