# Supplementary material for: Linking diet to growth, nutrient composition, and flavor characteristics in Chinese mitten crab (Eriocheir sinensis): a study based on biochemical composition and intestinal microbiota
Source: Front Nutr. 2026 Apr 1;13:1798709. doi: 10.3389/fnut.2026.1798709 (PMC13082253; doi:10.3389/fnut.2026.1798709)
Supplement: Supplementary file 4 [file Table_4.docx]

Table S4 Predicted functions of the intestinal microbiota of *E sinensis* fed on CF, FTF, HM and BL in KEGG level 2.

| Pathway level2 | BL+CF | CF | FTF+CF | HM+CF |
| --- | --- | --- | --- | --- |
| Carbohydrate metabolism | 0.088269 | 0.095639 | 0.090179 | 0.087598 |
| Lipid metabolism | 0.028826 | 0.028472 | 0.032045 | 0.027367 |
| Metabolism of cofactors and vitamins | 0.035635 | 0.034954 | 0.03493 | 0.035454 |
| Energy metabolism | 0.036547 | 0.037054 | 0.038305 | 0.036621 |
| Amino acid metabolism | 0.069207 | 0.068243 | 0.076224 | 0.073011 |
| Nucleotide metabolism | 0.021677 | 0.022757 | 0.019896 | 0.02208 |
| Biosynthesis of other secondary metabolites | 0.011492 | 0.012051 | 0.01276 | 0.012317 |
| Metabolism of terpenoids and polyketides | 0.011318 | 0.012124 | 0.013859 | 0.012315 |
| Xenobiotics biodegradation and metabolism | 0.022163 | 0.022796 | 0.029892 | 0.021713 |
| Metabolism of other amino acids | 0.01472 | 0.014974 | 0.016003 | 0.014997 |
| Glycan biosynthesis and metabolism | 0.007873 | 0.008144 | 0.006667 | 0.007685 |
| Translation | 0.013186 | 0.014645 | 0.012919 | 0.014035 |
| Chemical structure transformation maps | 5.39E-05 | 7.18E-05 | 0.000138 | 5.99E-05 |
| Global and overview maps | 0.351765 | 0.358469 | 0.367921 | 0.358326 |
| Drug resistance: Antimicrobial | 0.013618 | 0.012066 | 0.010305 | 0.012876 |
| Drug resistance: Antineoplastic | 0.002258 | 0.002356 | 0.00284 | 0.002293 |
| Membrane transport | 0.057967 | 0.064482 | 0.059233 | 0.058317 |
| Signal transduction | 0.0618 | 0.052728 | 0.045419 | 0.056987 |
| Cellular community - prokaryotes | 0.059368 | 0.051589 | 0.046213 | 0.055709 |
| Cell motility | 0.016977 | 0.01375 | 0.012033 | 0.01563 |
| Folding, sorting and degradation | 0.01156 | 0.010865 | 0.008944 | 0.011974 |
| Transcription | 0.000832 | 0.000905 | 0.000816 | 0.000947 |
| Replication and repair | 0.014284 | 0.015232 | 0.013275 | 0.014683 |
| Endocrine system | 0.005042 | 0.005216 | 0.005629 | 0.004866 |
| Signaling molecules and interaction | 0.00012 | 0.000123 | 0.000103 | 8.42E-05 |
| Cell growth and death | 0.010615 | 0.009713 | 0.00983 | 0.010058 |
| Transport and catabolism | 0.002496 | 0.002428 | 0.002722 | 0.002478 |
| Aging | 0.002654 | 0.002618 | 0.002392 | 0.002594 |
| Circulatory system | 0.000412 | 0.000349 | 0.000388 | 0.000394 |
| Development | 0 | 0 | 3.46E-07 | 0 |
| Immune system | 0.001568 | 0.00131 | 0.001123 | 0.001539 |
| Environmental adaptation | 0.002795 | 0.002501 | 0.002606 | 0.002718 |
| Nervous system | 0.001122 | 0.001161 | 0.001303 | 0.001507 |
| Endocrine and metabolic diseases | 0.001579 | 0.001575 | 0.001707 | 0.00164 |
| Excretory system | 0.000185 | 0.000184 | 0.000202 | 0.000179 |
| Digestive system | 0.000627 | 0.000616 | 0.00046 | 0.000546 |
| Neurodegenerative diseases | 0.003133 | 0.002645 | 0.003206 | 0.002935 |
| Substance dependence | 0.000194 | 0.000327 | 0.000425 | 0.000337 |
| Infectious diseases: Bacterial | 0.006523 | 0.005702 | 0.005155 | 0.005945 |
| Infectious diseases: Parasitic | 0.000615 | 0.000502 | 0.000608 | 0.000521 |
| Infectious diseases: Viral | 0.000989 | 0.001033 | 0.001924 | 0.000875 |
| Cancers: Overview | 0.004093 | 0.004088 | 0.004855 | 0.003995 |
| Cancers: Specific types | 0.001561 | 0.001492 | 0.002185 | 0.001562 |
| Immune diseases | 0.00036 | 0.000381 | 0.000271 | 0.000383 |
| Cardiovascular diseases | 0.001921 | 0.001667 | 0.002093 | 0.001852 |
